# Excessive Lipid Peroxidation in Uterine Epithelium Causes Implantation Failure and Pregnancy Loss

**DOI:** 10.1002/advs.202302887

**Published:** 2023-12-03

**Authors:** Yafang Lu, Yuhan Shao, Weiwei Cui, Zhaoyu Jia, Qian Zhang, Qing Zhao, Zi‐Jiang Chen, Junhao Yan, Bo Chu, Jia Yuan

**Affiliations:** ^1^ Advanced Medical Research Institute Cheeloo College of Medicine Shandong University Jinan Shandong 250012 China; ^2^ Center for Reproductive Medicine Shandong University Jinan Shandong 250021 China; ^3^ Key Laboratory of Reproductive Endocrinology of Ministry of Education Shandong University Jinan Shandong 250021 China; ^4^ Department of Cell Biology School of Basic Medical Sciences Cheeloo College of Medicine Shandong University Jinan Shandong 250012 China

**Keywords:** embryo implantation, female fertility, GPX4, lipid peroxidation, uterine receptivity

## Abstract

The uterine epithelium undergoes a dramatic spatiotemporal transformation to enter a receptive state, involving a complex interaction between ovarian hormones and signals from stromal and epithelial cells. Redox homeostasis is critical for cellular physiological steady state; emerging evidence reveals that excessive lipid peroxides derail redox homeostasis, causing various diseases. However, the role of redox homeostasis in early pregnancy remains largely unknown. It is found that uterine deletion of Glutathione peroxidase 4 (GPX4), a key factor in repairing oxidative damage to lipids, confers defective implantation, leading to infertility. To further pinpoint *Gpx4*’s role in different cell types, uterine epithelial‐specific *Gpx4* is deleted by a lactotransferrin *(Ltf)*‐*Cre* driver; the resultant females are infertile, suggesting increased lipid peroxidation levels in uterine epithelium compromises receptivity and implantation. Lipid peroxidation inhibitor administration failed to rescue implantation due to carbonylation of major receptive‐related proteins underlying high lipid reactive oxygen species. Intriguingly, superimposition of Acyl‐CoA synthetase long‐chain family member 4 (ACSL4), an enzyme that promotes biosynthesis of phospholipid hydroperoxides, along with uterine epithelial GPX4 deletion, preserves reproductive capacity. This study reveals the pernicious impact of unbalanced redox signaling on embryo implantation and suggests the obliteration of lipid peroxides as a possible therapeutic approach to prevent implantation defects.

## Graphical abstract

Aberrations or defects in the receptive and functional uterine epithelium result in implantation failure and compromise pregnancy outcomes. This work reports that excessive lipid peroxides in the GPX4‐deleted uterine epithelium cause pregnancy loss and female infertility. Administering lipid peroxidation inhibitors doesn't rescue implantation, but preserving ACSL4 alongside GPX4 deletion can be targeted to rebalance the redox system and restore reproductive capacity.

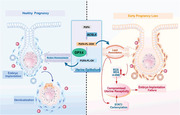

## 1 Introduction

A reciprocal interaction between a receptive uterus and an implantation‐competent blastocyst is the prerequisite for implantation success.^[^ ^1^, ^2^ ^]^ As the first maternal contact for embryos, the uterine epithelium undergoes dramatic molecular, physiological, and morphological changes to transmit embryonic signals to underlying stromal cells and initiate implantation. In mice, epithelial cells sustain proliferation influenced by preovulatory estrogen secretion on day 1 (day 1 = vaginal plug), followed by epithelial cell death on day 2 due to estrogen decline. Increasing progesterone (P_4_) levels from the newly formed corpora lutea on day 3 suspend epithelial cell death and trigger stromal cell proliferation. Epithelial cell proliferation ceases on day 4, achieving the receptive state and directing embryo apposition and adhesion.^[^ ^1^, ^3^, ^4^ ^]^ Numerous human studies also indicate that epithelial proliferation and death during the menstrual cycle require the interplay of ovarian hormones, transcriptional factors, cytokines, and other molecular signaling pathways.^[^ ^1^, ^4^, ^5^ ^]^ Aberrations or defects in the receptive and functional epithelium result in implantation failure and compromise pregnancy outcomes. Nevertheless, the hierarchical directives orchestrating uterine epithelium remodeling are still poorly understood.

Overt oxidative stress confers adverse effects on pregnancy.^[^ ^6^ ^]^ Lipid peroxidation is an oxidative damage process under which free radicals attack phospholipids (PLs) containing polyunsaturated fatty acids (PUFA) of the membranes of cellular or subcellular components. This process derails cellular redox homeostasis and is involved in diverse pathological progressions.^[^ ^7^, ^8^, ^9^ ^]^ Excessive lipid peroxides in certain cell types are accompanied by cell death and impaired integrity of organic morphology; based upon observations and expanded studies, lipid peroxidation has been proposed as the hallmark of ferroptosis, an iron‐dependent programmed cell death.^[^ ^9^, ^10^, ^11^, ^12^ ^]^ Nonetheless, the complexities of lipid peroxidation are context‐ and tissue‐dependent, in which some cell types retain cell proliferation and differentiation depending on particular cellular metabolic environments and repair abilities.

Glutathione peroxidase 4 (GPX4) is a highly evolutionarily conserved enzyme that acts as a phospholipid hydroperoxidase utilizing reduced glutathione (GSH) to convert phospholipid hydroperoxides (PL─OOH) to phospholipid alcohols (PL─OH), which serves to regulate lipid peroxide levels.^[^ ^8^, ^13^ ^]^ Depletion of GSH or inactivation of *GPX4* in cells leads to excessive reactive oxygen species (ROS) induced lipid peroxides and compromises redox homeostasis. *Gpx4*‐null mice show embryonic lethality due to intrauterine resorption around embryonic day 7.5.^[^ ^14^ ^]^ Studies in the past decade verify a broad requirement for *GPX4* in the maintenance of various cellular physiology, such as kidney, brain, liver, and immune responses.^[^ ^15^, ^16^, ^17^, ^18^, ^19^ ^]^ Evidence suggests that adverse effects of uncoordinated oxidative reactions caused by *GPX4* dysfunction can be blocked either by lipid ROS scavengers, or by inhibition of Acyl‐CoA synthetase long‐chain family member 4 (ACSL4) intercepting biosynthesis of PUFA‐containing phospholipids (PUFA‐PLs).^[^ ^20^, ^21^, ^22^ ^]^ These findings illustrate that the damaged redox network is pertinent and maneuverable for disease treatment.

Here we show that *Gpx4* is uniquely expressed in early pregnancy stages. Mice with uterine deletion of *Gpx4* by a progesterone receptor (*Pgr)‐Cre* driver (*Gpx4^f/f^Pgr^Cre^*) show embryo implantation failure resulting in complete infertility, despite normal uterine morphology and structure. Furthermore, uterine epithelial‐specific *Gpx4* deletion in adult mice by *Ltf‐Cre* (*Gpx4^f/f^Ltf^Cre^*) recapitulated the infertility phenotype. We also found that *Gpx4* deficiency increases lipid peroxidation levels, indicating that *Gpx4* plays a critical role in the epithelium for establishing uterine receptivity. Interestingly, lipid peroxidation inhibitor Liproxstatin‐1 (Lip1) administration failed to rescue fertility loss despite effectively attenuating lipid peroxidation levels in the uterus, which are associated with vital receptive‐related protein carbonylation. Consecutively, we introduced *Acsl4* deletion alongside *Gpx4* deletion in uterine epithelia (*Gpx4^f/f^Acsl4^f/f^Ltf^Cre^*) that were genetically predisposed to decrease lipid peroxidation levels; we observed full‐term pups from *Gpx4^f/f^Acsl4^f/f^Ltf^Cre^* females, although the litter sizes were significantly lower than littermate floxed mice. More importantly, we identified the conserved role of lipid peroxidation in the endometrium of mice and humans during early pregnancy. Our work provides evidence that elevating lipid peroxidation levels in the uterine epithelium severely compromises uterine receptivity and results in pregnancy loss.

## 2 Results

### 2.1 Uterine *Gpx4* Deletion Results in Complete Infertility With Failed or Defective Embryo Implantation

**Figure 1:**
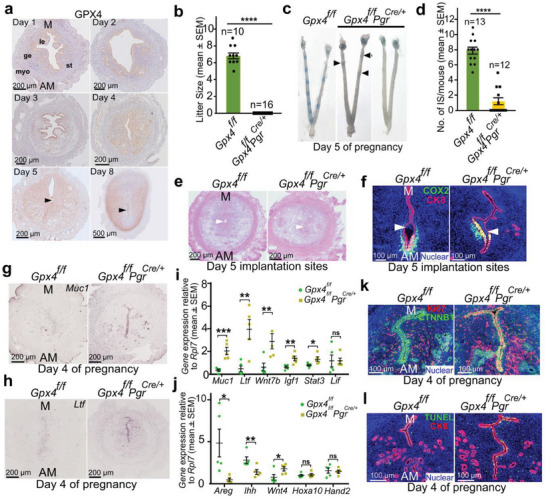
Uterine inactivation of *Gpx4* confers female infertility due to compromised uterine receptivity. a) IHC localization of GPX4 expression patterns on days 1–5 and 8 of pregnancy. Arrowheads point to the location of embryos. Scale bars, 200 µm (days 1–5) and 500 µm (day 8). M, mesometrial pole; AM, antimesometrial pole. le, luminal epithelium; ge, glandular epithelium; st, stroma; myo, myometrium. b) Pregnancy outcomes in *Gpx4^f/f^* and *Gpx4^f/f^Pgr^Cre/+^* mice. *Gpx4^f/f^Pgr^Cre/+^* females are completely infertile. The number above the bar indicates the number of mice tested. c) Day 5 implantation sites (blue bands) in *Gpx4^f/f^* and *Gpx4^f/f^Pgr^Cre/+^* females, arrows indicate weak blue bands. d) The average number of implantation sites per mouse from *Gpx4^f/f^* and *Gpx4^f/f^Pgr^Cre/+^* females on day 5 (n = 12–13 mice per group). Pregnancy of *Gpx4^f/f^Pgr^Cre/+^* females was confirmed by flushed embryos. e) Histology of day 5 implantation sites in *Gpx4^f/f^* and *Gpx4^f/f^Pgr^Cre/+^* mice. Arrowheads indicate the location of the embryos. Scale bars, 200 µm. f) IF of COX2 and CK8 in day 5 implantation sites of *Gpx4^f/f^* and *Gpx4^f/f^Pgr^Cre/+^* mice. Arrowheads indicate the location of the embryos. Scale bars, 100 µm. g,h) In situ hybridization of *Muc1* (g) and *Ltf* (h) in day 4 pregnant uteri from *Gpx4^f/f^* and *Gpx4^f/f^Pgr^Cre/+^* mice. Scale bars, 200 µm. i,j) Quantitative real‐time PCR analysis of receptivity marker genes (E_2_‐responsive genes (i) and P_4_‐responsive genes (j)) reveals impaired uterine receptivity in *Gpx4^f/f^Pgr^Cre/+^* mice on day 4 of pregnancy (n = 5 mice per group). k) IF of Ki67 and CTNNB1 in *Gpx4^f/f^* and *Gpx4^f/f^Pgr^Cre/+^* uteri on day 4 of pregnancy. Scale bars, 100 µm. l) IF of TUNEL and CK8 in *Gpx4^f/f^* and *Gpx4^f/f^Pgr^Cre/+^* uteri on day 4 of pregnancy. Scale bars, 100 µm. In (b), (d), (i), and (j), data are presented as mean ± SEM. ^*^ *p* < 0.05, ^**^ *p* < 0.01, ^***^ *p* < 0.001 and ^****^ *p* < 0.0001, ns: not significant, by two‐tailed Student's *t*‐test.

GPxs belong to a large family. Our previous RNA‐sequencing data^[^ ^23^ ^]^ in the isolated uterine epithelium and stromal cells from mice on day 4 of pregnancy show that *Gpx4* is one of the most abundant genes among the family members (Figure S1a, Supporting Information). To assess the spatiotemporal expression of *GPX4* during the peri‐implantation period, we performed immunohistochemistry (IHC) and found that *GPX4* was predominately localized in the epithelium from day 1 to day 4. *GPX4* signals were then observed primarily in stromal and decidual cells on day 5 and day 8 (**Figure** **1**a). In situ hybridization (ISH) of *Gpx4* showed a similar expression pattern in mRNA levels (Figure S1b, Supporting Information).

To explore the role of uterine *GPX4* in pregnancy, we generated females with uterine inactivation of *Gpx4* by crossing *Gpx4* floxed mice with *Cre* expression under the control of progesterone receptor transgenic mice (*Pgr‐Cre*).^[^ ^24^, ^25^ ^]^ These mice showed efficient deletion of *Gpx4* in the pregnant uterus at both mRNA and protein levels (Figure S1c–f, Supporting Information). We found total infertility in *Gpx4^f/f^Pgr^Cre/+^* females, as opposed to normal pregnancy in littermate *Gpx4^f/f^* females; both groups were mated with WT fertile males (Figure 1b). We detected the status of implantation sites on day 5 by blue dye injection,^[^ ^26^ ^]^ *Gpx4^f/f^Pgr^Cre/+^* females showed failed or defective embryo implantation compared with *Gpx4^f/f^* females (Figure 1c,d). This finding was reinforced by observations on day 5 of aberrant‐shaped crypt chambers, which are key to implantation success (Figure 1e),^[^ ^27^, ^28^ ^]^ along with altered expression of cyclooxygenase 2 (COX2), a marker for successful implantation (Figure 1f).^[^ ^29^ ^]^ These results show that *Gpx4* is critical for normal fertility. A careful examination of ovarian function was performed, because *Gpx4* is expressed in mouse ovary follicles,^[^ ^30^ ^]^ and *Pgr‐Cre* is expressed in ovaries.^[^ ^24^ ^]^ Serum Progesterone (P_4_) and 17β‐estradiol (E_2_) levels were comparable between *Gpx4^f/f^* and *Gpx4^f/f^Pgr^Cre/+^* mice on day 4 of pregnancy (Figure S2a,b, Supporting Information). Cytochrome p450 side‐chain cleavage enzyme (P450scc) and 3‐beta‐hydroxysteroid dehydrogenase (3β‐HSD), two key enzymes in P_4_ biosynthesis, showed no apparent changes in day 4 ovaries of *Gpx4^f/f^* and *Gpx4^f/f^Pgr^Cre/+^* mice (Figure S2c,d, Supporting Information). *Gpx4* deficiency may cause organ development issues due to ferroptosis;^[^ ^15^, ^18^ ^]^ in contrast, *Gpx4^f/f^Pgr^Cre/+^* females have normal ovarian and uterine structures and morphology, even though *Pgr‐Cre* is active in neonatal stages.^[^ ^28^ ^]^

### 2.2 Ablation of *Gpx4* in Uterus Compromises Uterine Receptivity

Following implantation failure, we then tested the uterine receptivity of *Gpx4^f/f^Pgr^Cre/+^* mice. Uterine expression of receptors for estrogen and progesterone (ESR1 and PR, respectively) showed no obvious alteration in *Gpx4^f/f^* and *Gpx4^f/f^Pgr^Cre/+^* uteri on day 4 of pregnancy (Figure S3a,b, Supporting Information). The expression pattern of FOXA2, a marker for gland function,^[^ ^31^ ^]^ also remained unaltered (Figure S3c, Supporting Information). However, ISH revealed the presence of mucin 1 (*Muc1)* and *Ltf*, two estrogen‐responsive epithelial genes, in day 4 uterine sections. Specifically, we found that *Muc1* and *Ltf* highly decorated the luminal epithelium in *Gpx4^f/f^Pgr^Cre/+^* uteri, as opposed to *Gpx4^f/f^* uteri (Figure 1g,h). We further analyzed the expression of genes known to be critical for uterine receptivity by Quantitative real‐time PCR (qRT‐PCR). The expression levels of E_2_‐responsive genes like *Muc1*, *Ltf*, Wnt family member 7B (*Wnt7b)*, insulin‐like growth factor 1 *(Igf1)*, and signal transducer and activator of transcription 3 (*Stat3)* were increased in day 4 uteri of *Gpx4* deleted mice, which indicated that inactivation of *Gpx4* confers increased estrogenic responses. Interestingly, as determined by leukemia inhibitory factor (Lif) expression, glandular function on day 4 uteri was comparable between floxed and deleted mice (Figure 1i). We further explored the expression of P_4_‐responsive genes: amphiregulin (*Areg)* and Indian hedgehog (*Ihh)* were decreased and Wnt family member 4 (*Wnt4)* was increased in *Gpx4^f/f^Pgr^Cre/+^* day 4 uteri, while homeobox A10 (*Hoxa10)* and heart and neural crest derivatives expressed 2 (*Hand2)* were unaltered (Figure 1j). The differential regulation of P_4_‐responsive genes suggests that depletion of *Gpx4* affects specific P_4_‐responsive genes in the uterus. These results demonstrate that compromised implantation stems from altered uterine sensitivity to hormone responsiveness.

The uterus must enter a receptive phase for successful implantation. One emphatic characteristic of uterine receptivity in mice is that luminal epithelial cells discontinue cell proliferation and cease cell death. Here, we observed the status of cell proliferation by performing immunofluorescence (IF) of the marker of proliferation Ki‐67 (Ki67) and phosphohistone H3 (pHH3) on day 4 uterine sections. We found that luminal epithelium in *Gpx4^f/f^Pgr^Cre/+^* day 4 uteri exhibited robust aberrant cell proliferation compared with floxed mice (Figure 1k; Figure S3d, Supporting Information); the result was also confirmed by cell proliferation markers cyclin D1 (*Ccnd1)*, minichromosome maintenance complex component 2 (*Mcm2)* and *Mcm7* by qRT‐PCR (Figure S3e, Supporting Information). Furthermore, we found unusual cell death in uterine epithelium of *Gpx4^f/f^Pgr^Cre/+^* uteri on day 4 of pregnancy by terminal deoxynucleotidyl transferase dUTP nick end labeling (TUNEL) staining (Figure 1l). However, cell death was not caused by apoptosis, confirmed by a lack of cleaved caspase‐3 (Cl‐*Casp3*) signaling in both floxed and deleted uteri (Figure S3f, Supporting Information). Relative genes for pyroptosis (*Casp11*, *Casp1*, and gasdermin D (*Gsdmd*))^[^ ^32^ ^]^ and ferroptosis (prostaglandin‐endoperoxide synthase 2 (*Ptgs2), Mdm2*, transferrin receptor (*Tfrc*), ferritin heavy chain 1 (*Fth*), arachidonate 12‐lipoxygenase (*Alox12*), and solute carrier family 7 member 11 (*Slc7a11*))^[^ ^11^, ^22^ ^]^ were analyzed by qRT‐PCR. The results validated increased cell death in *Gpx4^f/f^Pgr^Cre/+^* uteri on day 4 of pregnancy (Figure S3g,h, Supporting Information). Collectively, these results provide evidence that *Gpx4* deficiency compromises uterine receptivity perhaps due to dysfunctional luminal epithelia.

### 2.3 Adult Uterine Epithelium Inactivation of *Gpx4* is Sufficient to Compromise Embryo Implantation and Cause Infertility

**Figure 2:**
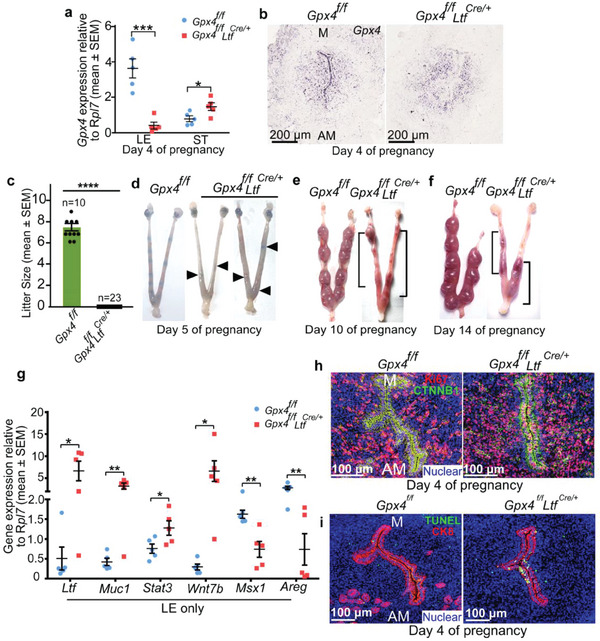
*Gpx4* specific deletion in the uterine epithelium results in female infertility due to failed or defective embryo implantation. a) Quantitative real‐time PCR of *Gpx4* in separated epithelial cells and stromal cells of *Gpx4^f/f^* and *Gpx4^f/f^Ltf^Cre/+^* mice on day 4 of pregnancy (n = 5 mice per group). LE, luminal epithelium; ST, stroma; b) In situ hybridization of *Gpx4* in day 4 uteri of *Gpx4^f/f^* and *Gpx4^f/f^Ltf^Cre/+^* mice. M, mesometrial pole; AM, antimesometrial pole. Scale bars, 200 µm. c) Pregnancy outcomes in *Gpx4^f/f^* and *Gpx4^f/f^Ltf^Cre/+^* mice. *Gpx4^f/f^Ltf^Cre/+^* females are completely infertile. The number above the bar indicates the number of mice tested. d–f) Representative uteri from *Gpx4^f/f^* and *Gpx4^f/f^Ltf^Cre/+^* mice on day 5 (d), day 10 (e), and day 14 (f). Bracket indicates adjacent resorption sites, arrowheads indicate weak blue bands. g) Quantitative real‐time PCR analysis of receptivity marker genes in separated epithelial cells reveals abnormal uterine receptivity in *Gpx4^f/f^Ltf^Cre/+^* mice on day 4 of pregnancy (n = 5 mice per group). h) IF of Ki67 and CTNNB1 in *Gpx4^f/f^* and *Gpx4^f/f^Ltf^Cre/+^* uteri on day 4 of pregnancy. Scale bars, 100 µm. i) IF of TUNEL and CK8 in *Gpx4^f/f^* and *Gpx4^f/f^Ltf^Cre/+^* uteri on day 4 of pregnancy. Scale bars, 100 µm. In (a), (c), and (g), data are presented as mean ± SEM. ^*^ *p* < 0.05, ^**^ *p* < 0.01, ^***^ *p* < 0.001 and ^****^ *p* < 0.0001, by two‐tailed Student's *t*‐test.

The *Pgr‐Cre* driver deletes gene expression in all major uterine cell types (myometrial, stromal, and epithelial cells).^[^ ^24^ ^]^ To further explore the specific contribution of epithelial *Gpx4* in uterine receptivity, we employed *Ltf‐Cre* driver, which is exclusively expressed in the uterine epithelium,^[^ ^33^ ^]^ to establish *Gpx4^f/f^Ltf^Cre/+^* mice with uterine epithelium‐selective *Gpx4* deletion. *Gpx4^f/f^Ltf^Cre/+^* mice showed efficient deletion of *Gpx4* in the uterine epithelium at both protein and mRNA levels (**Figure** **2**a,b; Figure S4a, Supporting Information). Interestingly, deletion of *Gpx4* in the epithelium causes increased *Gpx4* expression in stromal cells (Figure 2a). Pregnancy outcomes and litter size showed *Gpx4^f/f^Ltf^Cre/+^* females remain infertile (Figure 2c). A careful examination of fertility phenotypes showed that *Gpx4^f/f^Ltf^Cre/+^* females exhibited implantation failure on day 5 of pregnancy (Figure 2d) and complete fetal resorptions in the middle stage of pregnancy (Figure 2e,f).

To see whether uterine epithelial deletion of *Gpx4* gives rise to similar compromised uterine receptivity as seen in *Gpx4^f/f^Pgr^Cre/+^* females, we identified receptivity marker genes that are critical to uterine epithelial function in dissociated epithelial cells from day 4 of *Gpx4^f/f^* and *Gpx4^f/f^Ltf^Cre/+^* uteri. The results indicated an impaired balance between E_2_ and P_4_ responsiveness in uterine epithelium due to the absence of *Gpx4* (Figure 2g; Figure S4b, Supporting Information). Aberrant cell proliferation was also observed in the uterine epithelium of *Gpx4^f/f^Ltf^Cre/+^* mice on day 4 uteri by analysis of Ki67 and pHH3 staining (Figure 2h; Figure S4c, Supporting Information). In addition, anomalous uterine luminal epithelial cell death was detected as well (Figure 2i; Figure S4d,e, Supporting Information). Taken together, these data demonstrate that *Gpx4* in uterine epithelium contributes to epithelial remodeling for embryo implantation.

### 2.4 Redox Homeostasis of a Receptive Uterus is Disrupted Under Increased Lipid Peroxidation Levels in Mutant Mice

**Figure 3:**
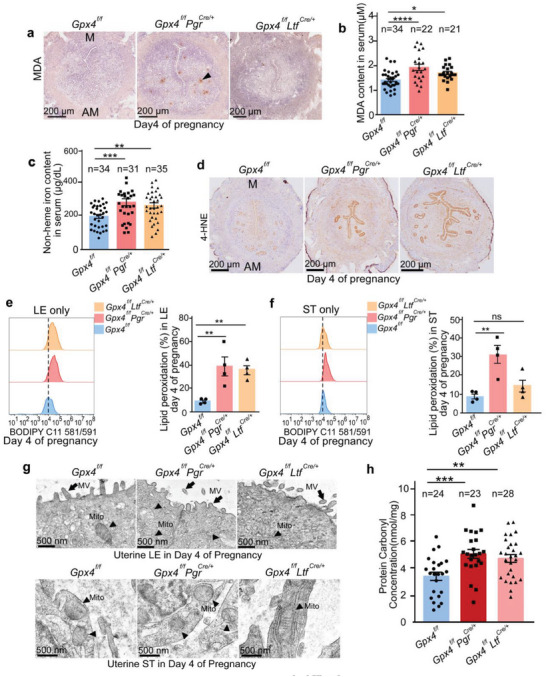
GPX4 deficiency increases the cellular level of lipid peroxides in the uterus on day 4 of pregnancy. a) IHC of malondialdehyde (MDA) in Gpx4^f^ *^/f^*, *Gpx4^f/f^Pgr^Cre/+^*, and *Gpx4^f/f^Ltf^Cre/+^* uteri on day 4 of pregnancy. M, mesometrial pole; AM, antimesometrial pole. Scale bars, 200 µm. Arrowhead indicates MDA signal. b) MDA levels in serum of *Gpx4^f/f^*, *Gpx4^f/f^Pgr^Cre/+^*, and *Gpx4^f/f^Ltf^Cre/+^* females on day 4 of pregnancy were measured according to MDA Assay Kit. The number above the bar indicates the number of mice tested. c) Non‐heme iron levels in serum of *Gpx4^f/f^*, *Gpx4^f/f^Pgr^Cre/+^*, and *Gpx4^f/f^Ltf^Cre/+^* females on day 4 of pregnancy were measured according to Iron Assay Kit. The number above the bar indicates the number of mice tested. d) IHC of 4‐Hydroxynonenal (4‐HNE) in *Gpx4^f/f^*, *Gpx4^f/f^Pgr^Cre/+^*, and *Gpx4^f/f^Ltf^Cre/+^* uteri on day 4 of pregnancy. Scale bars, 200 µm. e,f) Lipid peroxidation levels in separated uterine epithelial cells (e) and stromal cells (f) from *Gpx4^f/f^*, *Gpx4^f/f^Pgr^Cre/+^*, and *Gpx4^f/f^Ltf^Cre/+^* mice on day 4 of pregnancy were assessed with C11‐BODIPY 581/591 by flow cytometry (n = 4 mice per group). LE, luminal epithelium; ST, stroma; g) Representative transmission EM images of the apical surface of uterine luminal epithelial and stromal cell of *Gpx4^f/f^*, *Gpx4^f/f^Pgr^Cre/+^*, and *Gpx4^f/f^Ltf^Cre/+^* mice on day 4 of pregnancy. MV, microvilli; Mito, mitochondrion. LE, luminal epithelium; ST, stroma. Scale bar, 500 nm. h) Protein carbonylation levels in *Gpx4^f/f^*, *Gpx4^f/f^Pgr^Cre/+^*, and *Gpx4^f/f^Ltf^Cre/+^* uteri on day 4 of pregnancy were detected according to Protein Carbonyl ELISA Kit. The number above the bar indicates the number of mice tested. In (b‐c), (e‐f), and (h), data are reported as mean ± SEM. ^*^ *p* < 0.05, ^**^ *p* < 0.01, ^***^ *p* < 0.001 and ^****^ *p* < 0.0001, ns: not significant, by one‐way ANOVA followed by Dunnett's post hoc test.

GPX4 maintains cellular redox homeostasis by alleviating the accumulation of lipid hydroperoxides.^[^ ^12^, ^19^ ^]^ We speculated that uterine epithelia with *Gpx4* deletion are more susceptible to lipid peroxidation. Malondialdehyde (MDA) and 4‐hydroxynonenal (4‐HNE) are reactive aldehydes derived from lipid peroxidation initiated by non‐heme iron.^[^ ^34^, ^35^, ^36^ ^]^ Surprisingly, there is no MDA signaling on day 1, but a massive accumulation in the uterus was observed on day 2 of pregnancy in all mice types, and this surge gradually degraded from day 3 of pregnancy (Figure S5a, Supporting Information). Here, we assessed MDA accumulation in day 4 uterine sections. The results showed strong MDA concrescence in the stromal area of *Gpx4^f/f^Pgr^Cre/+^* females (**Figure** **3**a), with increased MDA signaling decorating the apical surface of *Gpx4* deleted uterine in both *Gpx4^f/f^Pgr^Cre/+^* and *Gpx4^f/f^Ltf^Cre/+^* females compared with floxed females (Figure S5b, Supporting Information). In the same context, the concentration of MDA and non‐heme iron in serum of day 4 pregnancy significantly increased in both *Gpx4* uterine mutant females compared to floxed females (Figure 3b,c). We then revealed that enhanced 4‐HNE was mainly retained in the uterine epithelium of both *Gpx4^f/f^Pgr^Cre/+^* and *Gpx4^f/f^Ltf^Cre/+^* females (Figure 3d).

To further illustrate the level changes of lipid peroxidation in uteri, we employed flow cytometry of Bodipy‐C11 581/591 probe (generally used to measure excessive phospholipid peroxidation in cell membranes) in isolated uterine epithelial and stromal cells from day 4 uteri.^[^ ^34^ ^]^ As indicated, obviously enhanced lipid peroxidation was detected in epithelial cells from both *Gpx4^f/f^Pgr^Cre/+^* and *Gpx4^f/f^Ltf^Cre/+^* females (Figure 3e). Lipid peroxidation also aggravated in stromal cells from *Gpx4^f/f^Pgr^Cre/+^* females. Conversely, no significant increased fluorescence intensities of Bodipy‐C11 581/591 were noted in stromal cells from *Gpx4^f/f^Ltf^Cre/+^* females (Figure 3f). Moreover, electron microscopy (EM) analysis exhibited aberrant mitochondrial morphology and reduction/vanishing of cristae structure in epithelial in both *Gpx4^f/f^Pgr^Cre/+^* and *Gpx4^f/f^Ltf^Cre/+^* day 4 uteri (Figure 3g). In addition, decreased and shortened microvilli on the apical surface of the epithelium were also observed in *Gpx4* mutant uteri (Figure S5c, Supporting Information). Although comparable mitochondrial morphology was observed in most stromal cells between *Gpx4^f/f+^* and *Gpx4^f/f^Ltf^Cre/+^* female (Figure 3g), it is important to note that mitochondrial abnormalities were found in stromal cells surrounding the epithelium in *Gpx4^f/f^Ltf^Cre/+^* mice (Figure S5d, Supporting Information). These results suggest that these stromal cells, located near the epithelium and known to be critical for implantation gland‐crypt assembly and primary decidual zone formation,^[^ ^37^ ^]^ may be affected by the bioactive lipid aldehydes released from *Gpx4*‐deficient epithelial cells. Our speculation that *Gpx4* ablation in the uterine epithelium is sensitive to lipid peroxidation was further confirmed by the finding of higher levels of protein carbonylation, a protein modification caused by lipid peroxidation‐derived aldehydes,^[^ ^38^, ^39^ ^]^ in *Gpx4* mutant uteri on day 4 of pregnancy (Figure 3h). These results suggest that *Gpx4* deletion increases lipid peroxidation levels in the uterine epithelium, conferring adverse effects that interfere with the homeostasis of epithelial‐stromal interactions required for achieving uterine receptivity and implantation.

### 2.5 Lipid ROS Attenuation by Lipid Peroxidation Inhibitor Lip1 in *Gpx4^f/f^Ltf^Cre^* Mice Failed to Rescue Implantation

**Figure 4:**
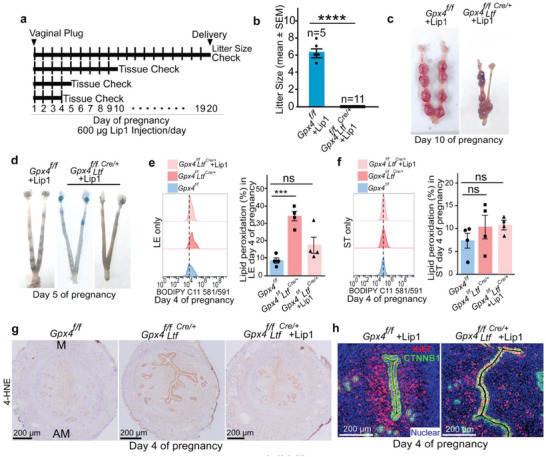
Lipid peroxidation inhibitor Liproxstatin‐1 (Lip1) administration in pregnant *Gpx4^f/f^Ltf^Cre/+^* mice fails to rescue implantation. a) Treatment schedule for Lip1. b) Pregnancy outcomes in *Gpx4^f/f^* and *Gpx4^f/f^Ltf^Cre/+^* mice after Lip1 treatment. The number above the bar indicates the number of mice tested. Data are presented as mean ± SEM. ^****^ *p* < 0.0001, by two‐tailed Student's *t*‐test. c,d) Representative uteri from *Gpx4^f/f^* and *Gpx4^f/f^Ltf^Cre/+^* mice on day 10 (c) and day 5 (d) after Lip1 treatment. e,f) Lipid peroxidation levels in separated uterine epithelial cells (e) and stromal cells (f) on day 4 after Lip1 treatment. Lipid peroxidation levels were comparable in separated uterine epithelial cells of *Gpx4^f/f^Ltf^Cre/+^* mice after Lip1 treatment with *Gpx4^f/f^* mice (n = 4 mice per group). LE, luminal epithelium; ST, stroma; In (e) and (f), data are reported as mean ± SEM. ^***^ *p* < 0.001, ns: not significant, by one‐way ANOVA followed by Dunnett's post hoc test. g) IHC of 4‐HNE in *Gpx4^f/f^*, *Gpx4^f/f^ Ltf^Cre/+^*, and *Gpx4^f/f^Ltf^Cre/+^* uteri after Lip1 treatment on day 4 of pregnancy. M, mesometrial pole; AM, antimesometrial pole. Scale bars, 200 µm. h) IF of Ki67 and CTNNB1 reveals LE aberrant proliferation in *Gpx4^f/f^Ltf^Cre/+^* uteri after Lip1 treatment on day 4 of pregnancy. Scale bars, 200 µm.

We next asked whether the attenuation of lipid peroxidation in the uterine epithelium rescues fertility loss. First, we established *GPX4* knockout clones in Ishikawa cells, a human endometrial epithelial cell line, using CRISPR‐Cas9 technology. As expected, increased lipid peroxidation levels were found in *GPX4* knockout subclones of Ishikawa cells (Figure S6a–c, Supporting Information), and lipid peroxidation inhibitor Liproxstatin‐1(Lip1) effectively alleviated lipid peroxidation in *GPX4* knockout Ishikawa cells (Figure S6d, Supporting Information). We then assessed the efficacy of Lip1 on pregnancy in *Gpx4^f/f^Ltf^Cre/+^* females. Lip1 was given by intraperitoneal injection (600 µg d^−1^) from day 1 of pregnancy in both *Gpx4^f/f^* and *Gpx4^f/f^Ltf^Cre/+^* females. Although the dosage of Lip1 did not appear to exert detrimental effects on pregnancy outcomes of *Gpx4^f/f^* females, it failed to rescue fertility in *Gpx4^f/f^Ltf^Cre/+^* females (**Figure** **4**a,b). We assessed the state of pregnancy on day 10 after Lip1 treatment and found degenerated implantation sites in *Gpx4^f/f^Ltf^Cre/+^* females (Figure 4c). By charting the initiation of adverse effects, we found that Lip1 failed to rescue implantation in *Gpx4^f/f^Ltf^Cre/+^* females (Figure 4d). To further determine whether Lip1 effectively diminishes lipid peroxidation in the uterus, Bodipy C11 581/591 staining was performed to evaluate lipid peroxidation levels in isolated epithelial and stromal cells from *Gpx4^f/f^* females and *Gpx4^f/f^Ltf^Cre/+^* females with or without Lip1 administration. The results demonstrated that Lip1 efficiently attenuated lipid peroxidation in epithelial cells from *Gpx4^f/f^Ltf^Cre/+^* day 4 uteri; Lipid peroxidation in stromal cells did not show further reduction (Figure 4e,f). 4‐HNE staining confirmed Lip1 administration competently alleviated lipid peroxidation in epithelial cells in *Gpx4^f/f^Ltf^Cre/+^* uteri (Figure 4g). Nevertheless, IF of Ki67 confirmed that abnormal cell proliferation persisted in *Gpx4^f/f^Ltf^Cre^* day 4 uteri after Lip1 treatment (Figure 4h).

**Figure 5:**
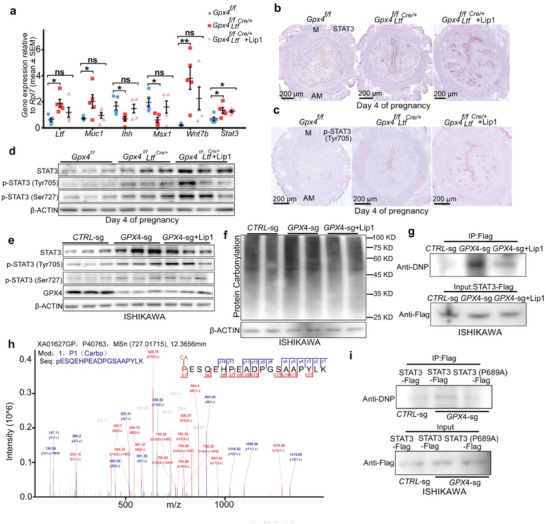
High lipid ROS influence STAT3 levels and activity in the uterine epithelium. a) Quantitative real‐time PCR analysis shows some receptivity marker genes were comparable in *Gpx4^f/f^Ltf^Cre/+^* mice after Lip1 treatment with *Gpx4^f/f^* mice. Data are mean ± SEM (n = 5 mice per group). ^*^ *p* < 0.05 and ^**^ *p* < 0.01, ns: not significant, by one‐way ANOVA followed by Dunnett's post hoc test. b,c) IHC of STAT3 (b) and p‐STAT3 (Tyr705) (c) in *Gpx4^f/f^*, *Gpx4^f/f^Ltf^Cre/+^*, and *Gpx4^f/f^Ltf^Cre/+^* uteri after Lip1 treatment on day 4 of pregnancy. Scale bars, 200 µm. d) Western blotting of STAT3, p‐STAT3 (Tyr705), p‐STAT3 (Ser727) in day 4 uteri of *Gpx4^f/f^*, *Gpx4^f/f^Ltf^Cre/+^*, and *Gpx4^f/f^Ltf^Cre/+^* after Lip1 treatment. e) Western blotting of STAT3, p‐STAT3 (Tyr705), p‐STAT3 (Ser727) in Ishikawa cells. f) DNP‐labeled carbonylated proteins in Ishikawa cells. β‐Actin was used as a loading control. g) DNP immunoblotting of STAT3‐Flag immunoprecipitated proteins in Ishikawa cells. h) LC–MS spectra of the carbonylation modification of STAT3 at residue Pro689. i) DNP immunoblotting of STAT3 (P689A)‐Flag immunoprecipitated proteins in GPX4 knockout Ishikawa cells.

We then detected receptivity‐related genes and found that some gene expression recovered to normal levels in *Gpx4^f/f^Ltf^Cre/+^* females after Lip1 administration compared with *Gpx4^f/f^* females, but partial genes retained aberrant expression (**Figure** **5**a). Similar results (Lip1 ameliorated lipid peroxidation elimination in uteri but failed to rescue compromised uterine receptivity) were also identified in *Gpx4^f/f^Pgr^Cre/+^* mice by Lip1 administration (Figure S7, Supporting Information). STAT3 is known to regulate epithelial estrogenic responses and epithelial remodeling during pregnancy, and mice with dysfunctional STAT3 in the uterine epithelium are infertile.^[^ ^40^, ^41^ ^]^ STAT3 retained abnormal expression in the uterine epithelium of *Gpx4^f/f^Ltf^Cre/+^* females after Lip1 administration, as evident from Western blotting and IHC results (Figure 5a–d). In vitro cell experiments showed similar abnormal STAT3 expression as in vivo (Figure 5e). Our previous results (Figure 3i) showed increased protein carbonylation levels in *Gpx4^f/f^Ltf^Cre/+^* day 4 uteri. Since protein carbonylation leads to inactivation of protein function,^[^ ^42^, ^43^ ^]^ we hypothesized that Lip1 administration failed to rescue implantation due to carbonylation of pivotal receptivity‐related proteins in *Gpx4* deleted uterine epithelium. We next examined the protein carbonylation levels in vitro and found increased carbonylation of proteins in *GPX4* knockout Ishikawa cells (Figure 5f). We then evaluated STAT3 carbonylation by performing immunoprecipitation experiments with transiently transfected STAT3 and blotted the immunoprecipitants with dinitrophenol (DNP) antibody in WT and *GPX4* knockout Ishikawa cells with or without Lip1 treatment, respectively. Increased levels of carbonylated STAT3 were detected in *GPX4* knockout Ishikawa cells even with Lip1 treatment (Figure 5g). Furthermore, we performed liquid chromatography–mass spectrometry (LC–MS) analysis using recombinant STAT3 protein in vitro with a carbonylation assay to determine the potential carbonylation sites of STAT3, identifying one carbonylation site, residue Pro689, on STAT3 (Figure 5h). STAT3 is an evolutionarily conserved gene and the residue P689 is also conserved across species. To confirm this carbonylation site, we constructed a carbonylation‐defective STAT3 mutant with proline residue substituted with alanine (P689A). STAT3 carbonylation was significantly reduced in STAT3 P689A ‐mutant‐transfected *GPX4* knockout Ishikawa cells (Figure 5i), which suggests that Pro689 is the essential residue for STAT3 carbonylation. These results demonstrate that the elimination of lipid peroxidation by Lip1 administration is insufficient to achieve completely functional uterine epithelial cells in early pregnancy due to the carbonylation of certain vital receptivity‐related proteins.

### 2.6 Deletion of *Acsl4* Contributes to Rescue Fertility Loss in Uterine Epithelial *Gpx4* Deficient Females

Based on the above observations, we next asked whether perturbation of various lipid peroxidation execution components could rescue *Gpx4* depletion in uterine epithelium mediated infertility. Acyl‐CoA synthetase long‐chain family member 4 (ACSL4), an enzyme that promotes biosynthesis of polyunsaturated phospholipids, acts as the main factor for lipid peroxidation. Recent reports show that ACSL4‐null cells are resistant to lipid peroxidation induced by inhibition or deactivation of *GPX4*.^[^ ^20^, ^44^ ^]^ We assessed the spatiotemporal uterine ACSL4 expression and revealed that ACSL4 is distinctly localized in luminal epithelial cells, with relatively lower levels in glandular epithelial cells before implantation; levels gradually elevate in decidual cells (Figure S8a,b, Supporting Information). Because of the prominent expression of ACSL4 in uterine epithelium, we speculated that inactivation of *Acsl4* may rescue infertility caused by *Gpx4* depletion in the uterine epithelium.

**Figure 6:**
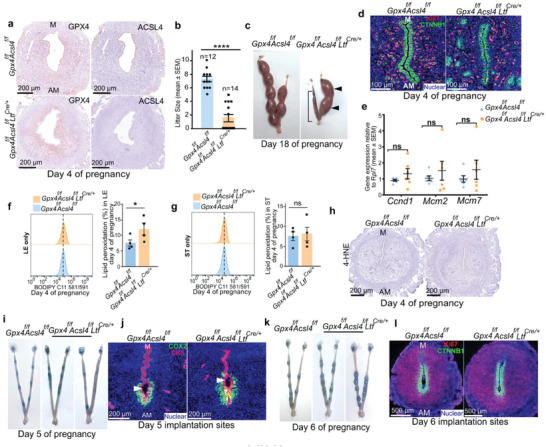
Superimposition of Acsl4 deletion on *Gpx4* uterine epithelial deficiency partially rescues implantation failure and preserves reproductive capacity. a) IHC of GPX4 and ACSL4 in serial sectioning of day 4 uteri of *Gpx4^f/f^Acsl4^f/f^* and *Gpx4^f/f^Acsl4^f/f^Ltf^Cre/+^* mice. Scale bars, 200 µm. b) Pregnancy outcomes in *Gpx4^f/f^Acsl4^f/f^* and *Gpx4^f/f^Acsl4^f/f^Ltf^Cre/+^* mice. The number above the bar indicates the number of mice tested. c) Representative uteri from *Gpx4^f/f^Acsl4^f/f^* and *Gpx4^f/f^Acsl4^f/f^Ltf^Cre/+^* mice on day 18. Bracket indicates adjacent resorption sites, arrowheads indicate viable fetuses. d,e) IF (d) of Ki67 and CTNNB1 and Quantitative real‐time PCR (e) of *Ccnd1, Mcm2, and Mcm7* reveal *Gpx4^f/f^Acsl4^f/f^Ltf^Cre/+^* mice have no aberrant epithelial cell proliferation on day 4 of pregnancy. Scale bars, 100 µm (n = 6 mice per group). f,g) Lipid peroxidation levels in separated uterine epithelial cells (f) and stromal cells (g) from *Gpx4^f/f^Acsl4^f/f^* and *Gpx4^f/f^Acsl4^f/f^Ltf^Cre/+^* mice on day 4 of pregnancy (n = 4 mice per group). LE, luminal epithelium; ST, stroma. h) IHC shows 4‐HNE were comparable in day 4 uteri of *Gpx4^f/f^Acsl4^f/f^* and *Gpx4^f/f^Acsl4^f/f^Ltf^Cre/+^* mice. Scale bars, 200 µm. i) Representative uteri from *Gpx4^f/f^Acsl4^f/f^* and *Gpx4^f/f^Acsl4^f/f^Ltf^Cre/+^* mice on day 5. j) IF of COX2 and CK8 in day 5 implantation sites of *Gpx4^f/f^Acsl4^f/f^* and *Gpx4^f/f^Acsl4^f/f^Ltf^Cre/+^* mice. Scale bars, 200 µm. Arrowheads indicate the location of the embryos. k) Representative uteri from *Gpx4^f/f^Acsl4^f/f^* and *Gpx4^f/f^Acsl4^f/f^Ltf^Cre/+^* mice on day 6. l) IF of Ki67 and CTNNB1 in day 6 implantation sites of *Gpx4^f/f^Acsl4^f/f^* and *Gpx4^f/f^Acsl4^f/f^Ltf^Cre/+^* mice. Scale bars, 500 µm. In (b,e–g), data are reported as mean ± SEM. ^*^ *p* < 0.05 and ^****^ *p* < 0.0001, ns: not significant, by two‐tailed Student's *t*‐test.

We first established uterine epithelial *Acsl4* deletion mice (*Acsl4^f/f^Ltf^Cre/+^*) (Figure S8b, Supporting Information). Intriguingly, *Acsl4^f/f^Ltf^Cre/+^* females did not exhibit any adverse reproductive phenotype (Figure S8c–f, Supporting Information). We then generated mice with conditional deletion of uterine epithelial *Gpx4* superimposed with deletion of *Acsl4* (*Gpx4^f/f^Acsl4^f/f^Ltf^Cre/+^*) by crossing *Gpx4^f/f^Ltf^Cre/+^* males with *Acsl4^f/f^* females (**Figure** **6**a). *Gpx4^f/f^Acsl4^f/f^Ltf^Cre/+^* females were mated with WT fertile males and produced a small number of pups (Figure 6b,c). We investigated the status of uterine receptivity of *Gpx4^f/f^Acsl4^f/f^Ltf^Cre/+^* females. Surprisingly, cell proliferation of *Gpx4^f/f^Acsl4^f/f^Ltf^Cre/+^* uteri was comparable with floxed uteri on day 4 of pregnancy (Figure 6d,e). Lipid peroxidation levels in isolated epithelial and stromal cells from *Gpx4^f/f^Acsl4^f/f^Ltf^Cre/+^* day 4 uteri were also evaluated, and epithelial cells of *Gpx4^f/f^Acsl4^f/f^Ltf^Cre/+^* females still displayed higher lipid peroxidation levels than floxed females (Figure 6f,g). Notably, the relative increase of lipid peroxidation levels of *Gpx4^f/f^Acsl4^f/f^Ltf^Cre/+^* females was much less than *Gpx4^f/f^Ltf^Cre/+^* females compared with floxed females. Identically, 4‐HNE seemed to impart no significant changes between double mutant mice and floxed mice by IHC (Figure 6h). qRT‐PCR showed that certain receptivity‐related genes retained aberrant expression (Figure S9a, Supporting Information), although STAT3 expression was comparable between floxed mice and mutant mice by IHC (Figure S9b,c, Supporting Information). All of the results support our hypothesis that deletion of *Acsl4* superimposed on uterine epithelial deletion of *Gpx4* ameliorates receptivity.

When examining the status of implantation sites on day 5, we often found strong blue bands, indicating improved embryo attachment in *Gpx4^f/f^Acsl4^f/f^Ltf^Cre/+^* females compared to *Gpx4^f/f^Ltf^Cre/+^* females (Figure 6i); implantation sites also had near‐normal crypt chamber shape and COX2 expression (Figure 6j). However, we consistently found the abnormal embryo distributions and different shades of blue bands on day 6 in *Gpx4^f/f^Acsl4^f/f^Ltf^Cre/+^* females (Figure 6k). As previously reported,^[^ ^37^ ^]^ stromal cells surrounding the implantation chamber in *Gpx4^f/f^Acsl4^f/f^* and *Gpx4^f/f^Acsl4^f/f^Ltf^Cre/+^* females were observed to transform into epithelial‐like cells to form the avascular primary decidual zone (PDZ), even though *Gpx4^f/f^Acsl4^f/f^Ltf^Cre/+^* females showed aberrant PDZ formation and abnormal decidualization (Figure 6l; Figure S9d,e, Supporting Information).

### 2.7 Decreased *GPX4* Expression in Endometrial Epithelium With High Lipid Peroxidation Levels is Evaluated in Recurrent Implantation Failure (RIF) Patients

**Figure 7:**
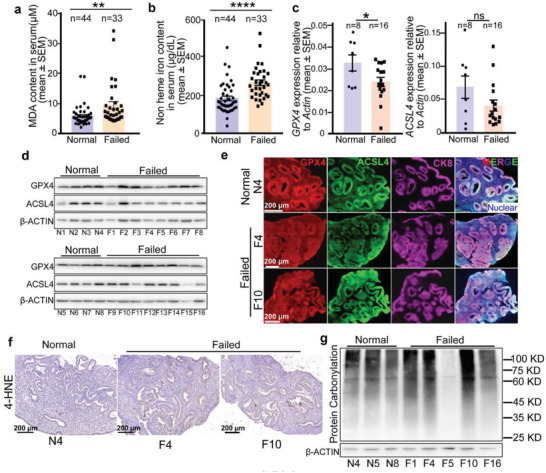
The decreased endometrial *GPX4* level is associated with increased lipid peroxide levels in recurrent implantation failure patients undergoing IVF treatment. a,b) MDA levels (a) and non‐heme iron levels (b) in serum indicate enhanced lipid peroxidation levels from women with recurrent implantation failure (n = 33) in comparison with successful pregnant women (n = 44). All the serum samples were collected one day before the participants embryo transplantation. c) Quantitative real‐time PCR analysis of *GPX4* and *ACSL4* from recurrent‐implantation‐failure patient endometrial tissues (n = 16) compared with those from a normal successful pregnancy (n = 8). d) Western blotting of GPX4 and ACSL4 protein levels in human endometrial tissues. β‐Actin was used as a loading control. e) IF localization of GPX4, ACSL4, and CK8 indicates an obviously decreased GPX4 expression in uterine epithelium of patients with recurrent implantation failure. CK8 served as an epithelial marker. Scale bars, 200 µm. f) IHC of 4‐HNE shows increased 4‐HNE in uterine epithelium of RIF patients. Scale bars, 200 µm. g) DNP‐labeled carbonylated proteins analysis in human endometrial tissues. β‐Actin was used as a loading control. In (a–c), data are presented as mean ± SEM. ^*^ *p* < 0.05, ^**^ *p* < 0.01 and ^****^ *p* < 0.0001, ns: not significant, by two‐tailed Student's *t*‐test.

We next investigated if increased lipid peroxidation levels or inhibition of *GPX4* is associated with human implantation disorders. We collected serum one day before embryo transplantation from participants: the control group sought in vitro fertilization‐embryo transfer (IVF‐ET) treatment because of ovulatory dysfunction, tubal factor, and male factors, and they eventually achieved successful pregnancy; the RIF group experienced recurrent implantation failure (at least 3 times), excluding those with ovulation disorders, chronic conditions like diabetes mellitus or hypertension, as well as hepatic and renal dysfunction. Patients from the two groups had comparable clinical parameters (Table S1, Supporting Information). We found MDA levels and non‐heme iron levels were significantly increased in the RIF group compared to the control group, indicating prominent lipid peroxidation in RIF patients (**Figure** **7**a,b).

We collected endometrial samples of the secretory phase (7 days after ovulation) from the normal menstrual cycle of both control participants with successful pregnancy after IVF‐ET and RIF participants (clinical parameters in Table S2, Supporting Information). We found the mRNA expression levels of *GPX4* were significantly lower in the RIF group than in the control group. In contrast, the mRNA levels of *ACSL4* showed unaltered expression between the two groups (Figure 7c). Decreased expression of the GPX4 protein was also validated in some RIF patients, especially in the endometrial epithelium of RIF patients. ACSL4 persisted with a comparable expression between the two groups (Figure 7d,e). Moreover, increased 4‐HNE levels confirmed high lipid peroxidation levels in the endometrial epithelium of RIF patients (Figure 7f). We also examined the protein carbonylation levels in human tissues, and found that individual RIF patients showed predominate protein carbonylation in the endometrium (Figure 7g). Overall, these novel findings suggest that aberrant endometrial *GPX4* expression with elevated levels of lipid peroxidation is one of the causes of recurrent implantation failure and infertility in women.

## 3 Conclusion and Discussion

Compromised redox homeostasis is associated with various physiologic and pathophysiologic processes. Previous studies have indicated that increased lipid peroxides are related to the pathogenesis of pregnancy‐relative diseases, such as preeclampsia and placental dysfunction.^[^ ^45^, ^46^, ^47^ ^]^ This study reveals an unidentified but pivotal role of lipid peroxidation in the receptive uterus before implantation initiation: redox signaling contributes to uterine epithelial transformation and remodeling to achieve a receptive physiological state.

Successful implantation requires effective bidirectional communication between the blastocyst and the receptive uterus.^[^ ^1^, ^2^ ^]^ Here, we demonstrate that uterine depletion of *Gpx4* (*Gpx4^f/f^Pgr^Cre^*) impedes receptivity and leads to female infertility. Notably, unlike dysplasia caused by *Gpx4* deficiency in some organs,^[^ ^15^, ^16^ ^]^ *Gpx4^f/f^Pgr^Cre^* mice show no structural defects in the female reproductive system, such as abnormal tissue size or poor adenogenesis, even though gene deletion in the uterus of *Pgr‐Cre* begins from a neonatal stage.^[^ ^28^ ^]^ In addition, *Pgr‐Cre* is not expressed in mouse embryos,^[^ ^27^, ^28^ ^]^ excluding the role of embryogenic *Gpx4* in this event, with gene expression deletion in both uterine epithelial and stromal cells. The paracrine secretions from stromal cells coordinate epithelial function.^[^ ^48^, ^49^ ^]^ By utilizing the *Ltf‐Cre* mouse line to create uterine epithelial‐specific *Gpx4* deletion (*Gpx4^f/f^Ltf^Cre^*) in mice, we confirmed that the absence of *Gpx4* in the uterine epithelium is sufficient to damage receptivity acquisition and confer pregnancy loss. These observations imply the indispensable role of *Gpx4* in the uterine epithelium to direct the establishment of implantation at peri‐implantation.

Although *GPX4* as a phospholipid hydroperoxidase governs lipid peroxidation by transducing toxic PL─OOH to nontoxic PL─OH and plays a critical role in ordering cellular redox homeostasis of organogenesis and tumorigenesis,^[^ ^9^, ^11^ ^]^ its function in implantation was unrecognized. Our findings of increased levels of MDA and 4‐HNE, two reactive toxic aldehydes decayed from lipid peroxides, in *Gpx4^f/f^Pgr^Cre^* and *Gpx4^f/f^Ltf^Cre^* day 4 uteri suggest that the redox signaling dominated by *GPX4* is a potential mechanism underlying this implantation process. This is also evident from the aberrant morphology of mitochondria in conjunction with increased lipid ROS in separated epithelial and stromal cells from *Gpx4* uterine mutant mice on day 4 of pregnancy. More noticeably, lipid peroxidation is normally supposed as a pathogenic factor,^[^ ^22^ ^]^ however, a surge of MDA accumulation was observed on day 2 and vanished on day 3 of pregnancy in wildtype mice, which was also confirmed in *Gpx4* mutant uteri during the pre‐receptive stage. This pattern is surprisingly synchronous and corresponds to the process of uterine epithelial apoptosis during the pre‐receptive stage in mice, emphasizing the importance of participation and coordination of lipid peroxidation in pre‐receptivity progress. The location of the surge of lipid peroxides in the human endometrium is not known, though a previous study indicated that redox balance is regulated by hormonal changes during the menstrual cycle.^[^ ^50^ ^]^ Progressively produced lipid peroxides may contribute to uterine epithelial transformation; one major function of *GPX4* in the uterus is to eliminate the surplus to assist the epithelium in achieving the receptive stage. This study uniquely reveals an unidentified role of *GPX4* in female fertility of mice, a role which is conserved in humans, as evidenced by the association between increased lipid peroxides and declining *GPX4* expression in the endometrium of women with recurrent implantation failure.

Liproxstatin‐1(Lip1) is a widely used lipid peroxidation inhibitor that has been reported to ameliorate ischemia/reperfusion‐induced tissue injury and alleviate acute renal failure resulting from *Gpx4* depletion in the kidney,^[^ ^16^ ^]^ which suggests Lip1 contributes to attenuating lipid peroxidation in vivo. It would be interesting to determine whether Lip1 administration could reduce lipid hydroperoxides in the uterus and rescue implantation failure. Nonetheless, our results provide evidence that Lip1 effectively reduced lipid peroxidation levels in the uterus without any observable adverse effects on fetal viability and growth, which, paradoxically, failed to improve embryo implantation. Notably, partial receptive‐relative gene expressions of *Gpx4* mutant uteri improved after Lip1 administration, indicating that certain receptive‐relative proteins are deprived of function due to irreversible modification underlying lipid ROS stress. Protein carbonylation resulting from ROS or downstream products of lipid peroxidation leads to protein dysfunction.^[^ ^39^, ^51^ ^]^ We identified STAT3, a key factor for uterine receptivity and embryo implantation,^[^ ^40^, ^41^ ^]^ as one of the carbonylated proteins. Consequently, Lip1 administration in relatively short time periods fails to reinstate uterine epithelial physiological function. Although the murine uterine epithelium undergoes cyclical turnover, there is evidence that regeneration and re‐expansion of luminal epithelial cells occur over several months, gradually populated by funder cells.^[^ ^52^ ^]^ Considering the reproductive life span of mice is significantly reduced at 7–8‐month‐old, long‐term treatment with a lipid peroxidation inhibitor to restore reproductive capacity in uterine epithelial *Gpx4* mutants may not be feasible. Nevertheless, evaluating the potential for a lipid peroxide cleaner to serve as a therapeutic approach for humans remains relevant, since the human endometrium undergoes dramatic remodeling, shedding, and regeneration during the menstrual cycle.

The observations of Lip1 administration inspired us to ask whether another site of action from the genetic origin could be targeted to rebalance the redox. We observed that ACSL4, a key player for lipid peroxidation involved in PL─OOH biosynthesis,^[^ ^20^, ^21^, ^22^ ^]^ shows abundant expression in the uterine epithelium at peri‐implantation. Interestingly, female mice with uterine epithelial ACSL4 deletion exhibit apparently normal pregnancy outcomes. Deletion of ACSL4 presents effective resistance to lipid peroxidation and ferroptosis caused by *GPX4* absence in vitro.^[^ ^20^, ^53^ ^]^ ACSL4 inactivation also confers protection against organ dysfunction due to lipid peroxides.^[^ ^54^, ^55^, ^56^ ^]^ We next asked whether uterine epithelium with ACSL4 inactivation displays robust resistance to lipid peroxidation caused by *GPX4* depletion to restore receptivity and fertility; we found that *Gpx4^f/f^Acsl4^f/f^Ltf^Cre^* females preserve reproductive capacity with compromised pregnancy outcomes. The epithelial cells of *Gpx4^f/f^Acsl4^f/f^Ltf^Cre^* receptive uteri retain mildly increased lipid peroxidation levels, which results in adverse ripple effects in implantation, decidualization, and placentation, indicating a hypersensitive response of receptive uterine epithelium to lipid peroxidation (Figure S10, Supporting Information).

Although lipid peroxides have been implicated in preeclampsia,^[^ ^46^, ^57^ ^]^ its perniciousness in early pregnancy remains unknown. Our mechanistic preclinical study addresses that anomalous lipid peroxidation in the uterine epithelium leads to implantation defects, encouraging clinical investigators to probe for enhanced lipid peroxides in women with recurrent implantation failure or recurrent spontaneous abortion. In the same vein, further investigating the role of lipid peroxidation in uterine stromal cells may deepen our understanding of endometrial redox homeostasis in implantation events. These findings raise the possibility of new therapeutic strategies to improve implantation rates by eliminating lipid ROS of uterus, thus improving the endometrial receptive environment.

## 4 Experimental Section

### Mice and Treatments

*Pgr^Cre/+^* mice were initially provided by John Lydon and Francesco DeMayo (Baylor College of Medicine, Houston, TX, USA),^[^ ^24^ ^]^ *Acsl4^f/f^* mice were generated in Wei Gu's lab (Columbia University, New York, NY, USA),^[^ ^44^ ^]^ *Ltf^Cre/+^* (#026030) and *Gpx4^f/f^* (#027964) mouse lines were purchased from Jackson Laboratories.^[^ ^25^, ^33^ ^]^ *Gpx4^f/f^Pgr^Cre/+^*, *Gpx4^f/f^Ltf^Cre/+^*, *Acsl4^f/f^Ltf^Cre^*, and *Gpx4^f/f^Acsl4^f/f^Ltf^Cre/+^* mice were generated by mating floxed females with *Pgr^Cre/+^* and *Ltf^Cre/+^* males,^[^ ^37^ ^]^ respectively. Pregnancy events were assessed as previously described.^[^ ^27^ ^]^ To assess pregnancy events, 8–12‐week‐old female mice were mated with fertile wildtype male mice to induce pregnancy (vaginal plug = day 1 of pregnancy). Pregnant females were sacrificed on day 5 by intravenous injection of Chicago blue dye to check the implantation sites. The litter size of pregnant mice was analyzed from day 17 to day 21 of pregnancy. For Liproxstatin‐1 (Selleck, S7699) treatment, each mouse was given an intraperitoneal injection of 600 µg at 9:00 a.m. from day 1 to the checkpoints of pregnancy.

### In Situ Hybridization

Frozen uterine sections of each genotype were processed onto the same slide and hybridized with a digoxin (DIG)‐labeled probe to observe the signal under the bright field of panoramic scanner (OLYMPUS, VS120). *Gpx4, Muc1, Ltf* probes were generated according to SP6/T7 Transcription Kit (Roche, 11175025910) and the linear template DNA was transcribed into digoxin‐labeled RNA by DIG‐RNA Labeling Mix (Roche, 11277073910). After RNA transcription, 1 µL of DNaseI (Biolabs, M0303S) was added to digest the DNA template. Then, 2 µL of 0.5 m EDTA, 2.5 µL of 4 m LiCl, and 75 µL of precooled ethanol were used to precipitate RNA overnight at −80 °C. Subsequently, RNA probes were purified, and the working concentration was generally 1 µg mL^−1^. The frozen sections were fixed in 4% PFAT (PFA+0.1% Tween 20 in PBS) at room temperature for 10 min, dehydrated and rehydrated with gradient methanol, followed by protease K (5 µg mL^−1^) digestion and acetylation. Anti‐DIG‐AP (Roche, 11093274910) was applied to hybrid slides sealed with blocking solution. The color reaction was observed by BCIP/NBT alkaline phosphatase color development kit (Roche,11697471001).

### Immunofluorescence

For immunofluorescence (IF) staining, frozen tissue was affixed to the specimen table using semi‐frozen OCT compound (Sakura, 4583) and then sliced at −20 °C using a low‐temperature freezing microtome (Leica, CM1860). Frozen sections (12 µm) from both the control and experimental groups were placed on the same slide and fixed in 4% paraformaldehyde (PFA) (Sigma, 158127‐500) for 10 min. Afterward, the sections were washed with 1× PBST (PBS+0.1% Triton X‐100) and blocked using a 5% bovine serum albumin (BSA) solution. Primary antibodies and corresponding secondary antibodies conjugated with Alexa 488 or Alexa 594 were then applied for visualization. Nuclear staining was carried out using DAPI (Solarbio, C0065, 5 µg mL^−1^). The TUNEL assay was performed using the TUNEL Apoptosis Assay Kit (Beyotime, C1086), following the manufacturer's instructions. Images were captured using a panoramic scanning fluorescence microscope (OLYMPUS, VS120) and processed using Oly VIA viewing software. The antibodies utilized in this study are listed in Table S3 (Supporting Information).

### Immunohistochemistry

Paraffin tissues for immunohistochemistry (IHC) were fixed in 10% neutral formalin, subjected to gradient ethanol dehydration treatment, and infiltrated with 0.1% Triton X‐100 (Solarbio, T8200) for 20 min. Subsequently, endogenous peroxidase activity was blocked using an endogenous peroxidase blocking solution (Beyotime, P0100A). The tissues were then treated with 5% BSA for 60 min to prevent non‐specific binding, followed by incubation with the appropriate antibodies. The chromogenic reaction was visualized using a DAB kit (Zhongshan Golden Bridge Bio‐technology, ZLI‐9018). The resulting images were captured and analyzed using a panoramic scanner bright field microscope (OLYMPUS, VS120).

### Lumen Epithelial Cells and Stromal Cells Separation

Mouse uterine lumen epithelial cells and stromal cells were isolated using a previously described method.^[^ ^27^ ^]^ The uterine tissue was extracted and cut into 2–3 mm fragments, which were then subjected to digestion at 37 °C for 1 h using pancreatin (Sigma, P3292) and dispase (Roche, 4942078001). Subsequently, the epithelial layer was carefully separated from the middle of the tissue fragments under a stereomicroscope, followed by rinsing with 1× PBS. The remaining tissue fragments were subjected to collagenase digestion (Sigma, C5138), and the resulting cell suspension was filtered through a nylon mesh to remove excess cell debris. After centrifugation, the isolated uterine stromal cells were collected.

### Quantitative RT‐PCR

Total RNA was extracted from uterine tissues using TRIzol reagent (Invitrogen, 15596‐018). 100 mg tissue was added with 1 mL of TRIzol and was homogenated on ice. Then 200 µL of chloroform was added to the sample, followed by vortexing and centrifugation at 12 000 rpm for 10 min at 4 °C. An equal volume of isopropanol was then added to the supernatant, mixed vigorously, left on ice for 30 min, and centrifuged again at 4 °C. 1 mL of 75% ethanol was used to wash the precipitate, and this procedure was repeated for RNA purification after centrifugation. 50 µL of RNase‐free water was added to dissolve the RNA. The RNA concentration and purity were measured using a UV–vis Spectrophotometer (Thermo, NanoDrop OneC), and gel electrophoresis was performed to ensure the quality of the RNA. 1 µg of RNA was used for reverse transcription into cDNA using an Evo M‐MLV RT Kit with a gDNA Clean Kit (Accurate Biology, AG11711). For every 1 µg of RNA, 1 µL of gDNA Clean Reagent, and 2 µL of the corresponding buffer were used to remove genomic DNA. RNase‐free water was then added to bring the volume up to 10 µL. The reaction was performed at 42 °C for 2 min using a PCR machine (BIO‐RAD, T100 Thermal Cycler), avoiding the influence of genomic DNA on the quantitative analysis of cDNA. The corresponding RT Kit was then used for reverse transcription, resulting in a total volume of 20 µL. The reaction conditions were set as follows: 37 °C for 15 min, 85 °C for 5 sec, and terminated at 4 °C. After diluting the sample fivefold with water, the cDNA diluent was used directly for quantitative PCR reactions. Quantitative real‐time PCR was performed with SYBR Green Master Mix (Vazyme, Q311‐02) and analyzed using a Real‐Time PCR system (Light Cycler 96) with specific primers listed in Table S4 (Supporting Information). 5 µL of cDNA template, 1 µL of the corresponding primer (4 µm), and 4 µL of RNase‐free water were combined to form a 20 µL reaction mixture. The relative mRNA level of each gene was calculated using the Δ*Ct* method with reference genes *Rpl7* and *β‐Actin*, which were used as internal controls in mouse and human tissues, respectively.

### Measurement of Serum P_4_ and E_2_ Levels

Sera were collected on day 4 of pregnancy. The concentration of P_4_ and E_2_ levels were measured by enzyme‐linked immunosorbent assay kit (Cayman,582601 and 501890, respectively.)

### Measurement of Serum Non‐Heme Iron Levels

This method was modified and combined according to published papers.^[^ ^58^, ^59^ ^]^ Briefly, 100 mg disodium‐4,7‐diphenyl‐1,10‐phenanthroline disulfonate (Solarbio, 52746‐49‐3) and 1.429 mL of 70% thioglycollic acid (Sigma, T6750) were dissolved in 60 mL distilled water to obtain iron chromogenic solution. To prepare the working solution, one volume of the iron coloration reservoir was added to five volumes of saturated sodium acetate solution and five volumes of distilled water. Eight standard substances in the concentration range of 0–1000 µg dL^−1^ were prepared according to instructions of iron standard solution (Sigma, 02583). 200 µL of iron working solution was added to 96‐well plates, followed by 10 µL serum and iron standard solution. The absorbance value was read by TECAN microplate reader (INFINITE 200 PRO) at 535 nm wavelength.

### Measurement of Serum MDA levels

This method was modified according to a published paper.^[^ ^60^ ^]^ 100 µL of the serum was added to 325 µL of 1‐methyl‐2‐phenylindole (Shanghai yuanye Bio‐Technology, S46375), then dissolved in an acetonitrile (Sigma–Aldrich,34851)‐methanol mixture (3:1, V/V), with a final concentration of 10 mm 1‐methyl‐2‐phenylindole. The reaction was then started by adding 75 µL of 37% hydrochloric acid. Upon incubation of the reaction mixture at 70 °C for 45 min, samples were centrifugated (10 min, 15 000 g, 4 °C) and the supernatant was collected. The 595 nm absorbance was measured, and the MDA concentration was determined against a standard of 1,1,3,3‐tetramethoxypropane (Sigma–Aldrich, 108383) as a source of MDA.

### FACS‐Based Lipid Peroxidation Assay

Cells were resuspended with PBS containing 5 µm C‐11 BODIPY 581/591 dye (Invitrogen, D3861) and incubated at room temperature for 30 min. Then, the cells were resuspended in 300 µL PBS and filtered through 0.45 µm nylon membrane into a sterile flow tube. ROS levels were analyzed by using flow cytometry (Gallios) through the FL1 channel. In each sample, 5000 cells were analyzed, and the data were analyzed using FlowJo.

### Measurement of Protein Carbonylation Levels

Protein carbonylation levels of mouse uterine tissues were detected by the OxiSelect Protein Carbonyl ELISA Kit (Cell Biolabs, STA‐310).

### Transmission Electron Microscopy

Fresh mouse uterine tissue was fixed in 2.5% glutaraldehyde/0.1 m phosphate buffer (pH 7.4) for 5 min. Subsequently, the tissue was cut into 1 mm^2^ sections and 2–3 mm length tissue blocks. These tissue blocks were then continuously fixed in the solution for 2 h at room temperature and subsequently treated overnight at 4 °C. The observed regions were located on the cross‐section of the tissue. Samples were rinsed with 0.1 m PBS and fixed with 1% osmic acid fixative (pH 7.4) for 2 h. After a 30‐min rinse with phosphate, the samples were treated with 30%, 50%, 70%, 90%, and 100% ethanol for 15 min each, followed by gradient dehydration using 100% acetone. The samples were then soaked, embedded in epoxy resin, and polymerized at 37, 45, and 60 °C for 12 h using an automatic tissue processor (Leica, EM TP). The tissue was subsequently cut into semi‐thin sections with a thickness of 5 µm using an ultramicrotome (Leica, EM UC7) and dried for 20 min. These sections were stained with toluidine blue (Sigma, 89640) and observed under a light microscope. Notably, the uterine lumen epithelium and stromal cells needed to be located within the same field of view. Once the semi‐thin sections were localized, the embedded blocks were further trimmed and sectioned with an ultra‐thin microtome to achieve a thickness of 50 nm. Ultrathin sections were stained with saturated aqueous uranyl acetate and lead citrate solution for 30 min in a dyeing machine (Leica, EM AC20). Subsequently, these sections were photographed and analyzed using the Thermo Fisher transmission electron microscope (Talos F200C).

### GPX4 Deleted Ishikawa Cells and Treatment

SgRNA of *GPX4* was constructed to pX458 and plenti‐CRISPR‐V2 vector. The sgRNA sequences were designed from the website (http://crispor.tefor.net/). sg*GPX4*: CACCGAGAGATCAAAGAGTTCGCCG, AAACCGGCGAACTCTTTGATCTCTC. Genomic deletions of *GPX4* in Ishikawa cells employing the CRISPR/Cas9 system were performed as previously described.^[^ ^61^ ^]^ The knockout efficiency was identified by western blotting. Ishikawa cells were cultured at 37 °C in an atmosphere of 5% CO_2_/95% air in DMEM‐F12 medium (M&C GENE, CM16405) supplemented with 10% (v/v) fetal bovine serum (Gibco, 10091148). Ishikawa cells were treated with Liproxstatin‐1 (Selleck, S7699) and Necrostatin‐1 (Selleck, S8037) at a concentration of 2 µm for 24 h, and z‐VAD‐FMK (Selleck, S7023) was treated with 20 µm. Subsequently, cells were collected to detect ROS levels.

### Immunoblotting

For immunoblot analysis, proteins from tissues or cells were extracted using RIPA buffer (Beyotime, P0013B), which was supplemented with 1% protease inhibitor (Roche, 04693132001) or phosphatase inhibitor cocktails (Roche, 04906845001). The protein concentration was determined using a Pierce BCA Protein Assay Kit (Thermo, 23227) to ensure equal loading of protein across different samples. Total proteins were separated through 10% SDS‐PAGE gel electrophoresis, followed by transfer onto a PVDF membrane (Millipore, ISEQ00011). After transfer, the membrane was blocked with 5% fat‐free milk for 1 h at room temperature and subsequently incubated with the indicated primary antibodies, followed by the corresponding secondary antibodies. β‐Actin served as a loading control. Bands were visualized using an ECL kit (Thermo,32106) and detected by the Amersham Imager 680 System (Chemiluminescence).

### Total Protein Carbonylation Assay

Carbonylated proteins from Ishikawa cells and patient endometrium samples were detected using the OxiSelect Protein Carbonyl Immunoblot Kit (Cell Biolabs, STA‐308). In brief, the proteins were separated through SDS‐PAGE and then transferred onto a PVDF membrane. The process of dinitrophenylhydrazine (DNPH) derivatization of oxidized proteins on the PVDF membrane was performed. This derivatization involved converting the carbonyl groups on the side chains of proteins into 2,4‐dinitrophenylhydrazones. After derivatization, the membrane was incubated with a horseradish peroxidase antibody conjugate against the anti‐2,4‐dinitrophenylhydrazone antibody. Following these steps, the carbonylated proteins were visualized and analyzed using immunoblotting techniques.

### Immunoprecipitation

Immunoprecipitation was performed as previously described.^[^ ^62^ ^]^ The plasmid expressing Stat3‐Flag was transfected into Ishikawa cells using Lipofectamine 3000 reagent (Invitrogen, L3000015). Protein supernatants were collected and incubated with 1 µg of anti‐Flag antibody (Sigma, F1804), followed by the addition of protein A/G beads (Santa Cruz, B2422). After an overnight incubation at 4 °C, the beads were washed four times using lysis buffer. The immunoprecipitated proteins were separated by SDS‐PAGE and analyzed by Western blot using anti‐Flag (Sigma, F1804) and DNP (Cell Biolabs) antibodies.

### Protein Identification and Quantification by LC–MS/MS Analyses

Follow the above procedure to collect supernatants incubated with flag antibodies and beads, separated immunoprecipitated proteins by SDS‐PAGE were stained with Coomassie Blue Fast Staining Solution (Beyotime, P0017). Target gel pieces were cut and destained in 50 mm NH_4_HCO_3_ in 50% acetonitrile (v/v) until clear. Gel pieces were dehydrated with 100 µL of 100% acetonitrile for 5 min, the liquid removed, and the gel pieces rehydrated in 10 mm dithiothreitol and incubated at 56 °C for 60 min. Gel pieces were again dehydrated in 100% acetonitrile, liquid was removed and gel pieces were rehydrated with 55 mM iodoacetamide. Samples were incubated at room temperature, in the dark for 45 min. Gel pieces were washed with 50 mm NH_4_HCO_3_ and dehydrated with 100% acetonitrile. Gel pieces were rehydrated with 10 ng µL^−1^ trypsin resuspended in 50 mm NH_4_HCO_3_ on ice for 1 h. Excess liquid was removed and gel pieces were digested with trypsin at 37 °C overnight. Peptides were extracted with 50% acetonitrile/5% formic acid, followed by 100% acetonitrile. Peptides were dried to completion and resuspended in 2% acetonitrile/0.1% formic acid. After trypsin digestion, the tryptic peptides were separated at a constant flowrate of 400 nL min^−1^ on an EASY‐nLC 1000 UPLC system (Thermo). The peptides were subjected to NSI source followed by tandem mass spectrometry (MS/MS) in Q Exactive Plus (Thermo) coupled online to the UPLC. The electrospray voltage applied was 2.0 kV. The full MS scan resolution was set to 70 000 for a scan range of 350–1800 *m*/*z*. Peptides were then selected for MS/MS using NCE setting as 28 and the fragments were detected in the Orbitrap at a resolution of 17 500. A data‐dependent procedure alternated between one MS scan followed by 20 MS/MS scans with 15.0 s dynamic exclusion. Automatic gain control (AGC) was set at 5E4. The resulting MS/MS data were processed using Proteome Discoverer 1.3. The database was set as the target protein Stat3 sequence. Trypsin/P was specified as cleavage enzyme. The carbonylation modifications were set to 4 missing cleavages and mass tolerance for precursor ions was set as 10 ppm in main search, and mass tolerance for fragment ions was set as 0.02 Da. Peptide confidence was set at high, and peptide ion score was set >20.

### Human Serum Sample Collection

Female patients who were advised to choose in vitro fertilization or intracytoplasmic sperm injection (IVF/ICSI) treatment at the Center for Reproductive Medicine, Shandong Provincial Hospital Affiliated with Shandong University were recruited. All the serum samples were collected one day prior to participant embryo transfer. Normal control serum from 44 cycles were collected from patients whose indications for IVF/ICSI included ovulatory dysfunction, tubal factor, and male factors, and who had achieved successful pregnancy after embryo transfer. For collection of serum samples in women who had experienced cumulative recurrent implantation failure (RIF), only patients who had undergone at least 3 in vitro fertilization‐embryo transfer (IVF‐ET) failures, in which no less than 4–6 eight‐cell embryos of high scores or three high‐quality blastocysts were transferred in total, were included. Furthermore, all 33 recruited patients in the RIF group experienced implantation failure after the embryo transfer. A detailed description of patient clinical parameters is listed in Table S1 (Supporting Information). All patients in these two groups had good basal hormonal levels and a good response to hormonal stimulation (>8 oocytes/ oocyte retrieval). Each serum sample was collected by centrifugation (3500 rpm, 10 min) of peripheral blood and frozen at −80 °C until detection.

### Endometrial Sample Collection

Endometrial biopsies were collected using a Pipelle de Cornier device on day 7 after ovulation (Ovulation Day was determined by B‐mode ultrasound). All participants had undergone at least two to three IVF‐ET failures, in which no less than 4–6 eight‐cell embryos of high scores or two high‐quality blastocysts were transferred in total. Participants who received hormonal therapy during the last three menstrual cycles were excluded. The pregnancy outcomes of the next transfer cycles following the endometrial biopsies determined the successfully implanted group and implantation failure group. The criteria of successful implantation were the detection of a gestational sac by B‐mode ultrasound. Each tissue sample was divided into two pieces and immediately frozen in liquid nitrogen or fixed in 4% formalin for immunohistochemical analyses.

### Multiplex Immunofluorescence and Image Analysis

Three‐color multiplex immunofluorescence was performed with the OPAL Polaris system (Akoya Biosciences) for Gpx4 (1: 100), Acsl4 (1: 200), and CK8 (1: 500) on slices. In short, slices were baked for 2 h at 60 °C, then dehydrated and hydrated. All slides were performed with heat‐induced epitope retrieval in Tris‐EDTA buffer (pH9.0) for 15 min, then washed with 1× TBST buffer (Solarbio, T1082) and blocked non‐specific binding with 30% goat serum for 1 h. According to the manufacturer's instructions, primary antibodies were diluted with Opal Antibody Diluent (ARD1001EA) and applied at 4 °C overnight. The slices were incubated for 10 min with Oppl Polymer HRP Anti‐Mouse/Rabbit (ARH1001EA), sequentially stained with tyramide signal amplification and OPAL fluorophore (NEL811001KT). Opal detector fluorophores were used for each marker as follows: OPAL 620 (*Gpx4*), 690 (Acsl4), and 520 (CK8) dye. The slides were counterstained using DAPI and sealed in Anti‐Fluorescence Mountain Medium (Abcam, ab104135). The stained slides were imaged and scanned using multispectral panoramic tissue scanning microscopy (TissueFAXS Spectra).

### Statistical Analysis

Statistical analyses were performed using three tests: a two‐tailed Student's *t*‐test to compare the means of two groups; one‐way ANOVA followed by Dunnett's post hoc test to compare the differences between the means of three independent groups; and the non‐parametric Kolmogorov–Smirnov test to compare the distributions of two unpaired groups, all conducted in Prism GraphPad software (version 8.3). Data for normally distributed variables were presented as the mean ± SD/SEM, and data for non‐normally distributed variables were presented as medians and quartiles, respectively. A significance level of *p* < 0.05 was considered statistically significant.

### Ethics Statement

All mice used in this study were housed in barrier facilities at Shandong University's Animal Care Facility according to the National Institutes of Health Guide for the Care and Use of Laboratory Animals, with the approval of the Institutional Animal Care and Use Committee of Shandong University (Permit No. ECSBMSSDU2022‐2‐68). Ethics approval for the collection and use of human serum and endometrial samples was authorized by the Ethics Committee Review Board of School of Basic Medical Sciences, Shandong University. All the recruited participants gave their written informed consent (Permit No. ECSBMSSDU2022‐1‐41).

## Conflict of Interest

The authors declare no conflict of interest.

## Author Contributions

Y.L., Y.S., and W.C. contributed equally to this work. Y.L., Y.S., and W.C. performed the experiments and analyzed the data; Y.S., Q.Z., Q.Z., Z.‐J.C., and J.Y. collected the human samples; Y.L. and Z.J. developed the conditional mouse model; Y.L., Y.S., J.Y., B.C., and J.Y. designed experiments; J.Y., B.C., and J.Y. supervised the study; Y.L., Y.S., and J.Y. wrote the manuscript. The order of the co–first authors was assigned on the basis of their relative contributions to the study.

## Supporting information

Supporting Information

## Data Availability

The data that support the findings of this study are available from the corresponding author upon reasonable request.

## References

[1] J. Cha , X. Sun , S. K. Dey , Nat. Med. 2012, 18, 1754. 23223073 10.1038/nm.3012 PMC6322836

[2] H. Wang , S. K. Dey , Nat. Rev. Genet. 2006, 7, 185. 16485018 10.1038/nrg1808

[3] Q. Zhang , B. C. Paria , Endocrinology 2006, 147, 2215. 16469810 10.1210/en.2005-1555 PMC1456201

[4] Q. Xin , S. Kong , J. Yan , J. Qiu , B. He , C. Zhou , Z. Ni , H. Bao , L. Huang , J. Lu , G. Xia , X. Liu , Z.‐J. Chen , C. Wang , H. Wang , J. Clin. Invest. 2018, 128, 175. 29202468 10.1172/JCI92862 PMC5749512

[5] C. Ifenatuoha , B. Okewale , Hum. Fertil. 2022, 25, 848. 10.1080/14647273.2021.1925976 34002655

[6] Y. Hirota , N. Acar , S. Tranguch , K. E. Burnum , H. Xie , A. Kodama , Y. Osuga , I. Ustunel , D. B. Friedman , R. M. Caprioli , T. Daikoku , S. K. Dey , Proc. Natl. Acad. Sci. 2010, 107, 15577. 20713718 10.1073/pnas.1009324107 PMC2932576

[7] H. Yin , L. Xu , N. A. Porter , Chem. Rev. 2011, 111, 5944. 21861450 10.1021/cr200084z

[8] G. Lei , L. Zhuang , B. Gan , Nat. Rev. Cancer 2022, 22, 381. 35338310 10.1038/s41568-022-00459-0 PMC10243716

[9] X. Jiang , B. R. Stockwell , M. Conrad , Nat. Rev. Mol. Cell Biol. 2021, 22, 266. 33495651 10.1038/s41580-020-00324-8 PMC8142022

[10] J. Zheng , M. Conrad , Cell Metab. 2020, 32, 920. 33217331 10.1016/j.cmet.2020.10.011

[11] D. Chen , B. Chu , X. Yang , Z. Liu , Y. Jin , N. Kon , R. Rabadan , X. Jiang , B. R. Stockwell , W. Gu , Nat. Commun. 2021, 12, 3644. 34131139 10.1038/s41467-021-23902-6 PMC8206155

[12] B. R. Stockwell , J. P. Friedmann Angeli , H. Bayir , A. I. Bush , M. Conrad , S. J. Dixon , S. Fulda , S. Gascón , S. K. Hatzios , V. E. Kagan , K. Noel , X. Jiang , A. Linkermann , M. E. Murphy , M. Overholtzer , A. Oyagi , G. C. Pagnussat , J. Park , Q. Ran , C. S. Rosenfeld , K. Salnikow , D. Tang , F. M. Torti , S. V. Torti , S. Toyokuni , K. A. Woerpel , D. D. Zhang , Cell 2017, 171, 273. 28985560 10.1016/j.cell.2017.09.021 PMC5685180

[13] G. C. Forcina , S. J. Dixon , Proteomics 2019, 19, e1800311. 30888116 10.1002/pmic.201800311

[14] L. J. Yant , Q. Ran , L. Rao , H. Van Remmen , T. Shibatani , J. G. Belter , L. Motta , A. Richardson , T. A. Prolla , Free Radic. Biol. Med. 2003, 34, 496. 12566075 10.1016/s0891-5849(02)01360-6

[15] B. A. Carlson , R. Tobe , E. Yefremova , P. A. Tsuji , V. J. Hoffmann , U. Schweizer , V. N. Gladyshev , D. L. Hatfield , M. Conrad , Redox Biol. 2016, 9, 22. 27262435 10.1016/j.redox.2016.05.003 PMC4900515

[16] J. P. Friedmann Angeli , M. Schneider , B. Proneth , Y. Y. Tyurina , V. A. Tyurin , V. J. Hammond , N. Herbach , M. Aichler , A. Walch , E. Eggenhofer , D. Basavarajappa , O. Rådmark , S. Kobayashi , T. Seibt , H. Beck , F. Neff , I. Esposito , R. Wanke , H. Förster , O. Yefremova , M. Heinrichmeyer , G. W. Bornkamm , E. K. Geissler , S. B. Thomas , B. R. Stockwell , V. B. O'donnell , V. E. Kagan , J. A. Schick , M. Conrad , Nat. Cell Biol. 2014, 16, 1180. 25402683 10.1038/ncb3064 PMC4894846

[17] M. Jia , D. Qin , C. Zhao , L. Chai , Z. Yu , W. Wang , L. Tong , L. Lv , Y. Wang , J. Rehwinkel , J. Yu , W. Zhao , Nat. Immunol. 2020, 21, 727. 32541831 10.1038/s41590-020-0699-0

[18] L. Chen , W. S. Hambright , R. Na , Q. Ran , J. Biol. Chem. 2015, 290, 28097. 26400084 10.1074/jbc.M115.680090 PMC4653669

[19] I. Ingold , C. Berndt , S. Schmitt , S. Doll , G. Poschmann , K. Buday , A. Roveri , X. Peng , F. Porto Freitas , T. Seibt , L. Mehr , M. Aichler , A. Walch , D. Lamp , M. Jastroch , S. Miyamoto , W. Wurst , F. Ursini , E. S. J. Arnér , N. Fradejas‐Villar , U. Schweizer , H. Zischka , J. P. Friedmann Angeli , M. Conrad , Cell 2018, 172, 409. 29290465 10.1016/j.cell.2017.11.048

[20] S. Doll , B. Proneth , Y. Y. Tyurina , E. Panzilius , S. Kobayashi , I. Ingold , M. Irmler , J. Beckers , M. Aichler , A. Walch , H. Prokisch , D. Trümbach , G. Mao , F. Qu , H. Bayir , J. Füllekrug , C. H. Scheel , W. Wurst , J. A. Schick , V. E. Kagan , J. P. F. Angeli , M. Conrad , Nat. Chem. Biol. 2017, 13, 91. 27842070 10.1038/nchembio.2239 PMC5610546

[21] B. Gan , Signal Transduct Target Ther. 2022, 7, 128. 35459217 10.1038/s41392-022-01004-z PMC9033814

[22] B. R. Stockwell , Cell 2022, 185, 2401. 35803244 10.1016/j.cell.2022.06.003 PMC9273022

[23] W. Deng , J. Yuan , J. Cha , X. Sun , A. Bartos , H. Yagita , Y. Hirota , S. K. Dey , Cell Rep. 2019, 27, 1755. 31067461 10.1016/j.celrep.2019.04.049 PMC6554729

[24] S. M. Soyal , A. Mukherjee , K. Y.‐S. Lee , J. Li , H. Li , F. J. Demayo , J. P. Lydon , Genesis. 2005, 41, 58. 15682389 10.1002/gene.20098

[25] S.‐E. Yoo , L. Chen , R. Na , Y. Liu , C. Rios , H. Van Remmen , A. Richardson , Q. Ran , Free Radic. Biol. Med. 2012, 52, 1820. 22401858 10.1016/j.freeradbiomed.2012.02.043 PMC3341497

[26] A. Psychoyos , Handbook of Physiology, American Physiology Society, Washington, D.C., USA 1973.

[27] J. Yuan , J. Cha , W. Deng , A. Bartos , X. Sun , H.‐Y. H. Ho , J.‐P. Borg , T. P. Yamaguchi , Y. Yang , S. K. Dey , Proc. Natl. Acad. Sci. 2016, 113, E8079. 27911818 10.1073/pnas.1614946113 PMC5167210

[28] J. Yuan , W. Deng , J. Cha , X. Sun , J.‐P. Borg , S. K. Dey , Nat. Commun. 2018, 9, 603. 29426931 10.1038/s41467-018-03092-4 PMC5807548

[29] H. Lim , B. C. Paria , S. K. Das , J. E. Dinchuk , R. Langenbach , J. M. Trzaskos , S. K. Dey , Cell 1997, 91, 197. 9346237 10.1016/s0092-8674(00)80402-x

[30] F. Wang , Y. Liu , F. Ni , J. Jin , Y. Wu , Y. Huang , X. Ye , X. Shen , Y. Ying , J. Chen , R. Chen , Y. Zhang , X. Sun , S. Wang , X. Xu , C. Chen , J. Guo , D. Zhang , Nat. Commun. 2022, 13, 5871. 36198708 10.1038/s41467-022-33323-8 PMC9534854

[31] A. M. Kelleher , J. Milano‐Foster , S. K. Behura , T. E. Spencer , Nat. Commun. 2018, 9, 2435. 29934619 10.1038/s41467-018-04848-8 PMC6015089

[32] P. Yu , X. Zhang , N. Liu , L. Tang , C. Peng , X. Chen , Signal Transduct Target Ther. 2021, 6, 128. 33776057 10.1038/s41392-021-00507-5 PMC8005494

[33] T. Daikoku , Y. Ogawa , J. Terakawa , A. Ogawa , T. Defalco , S. K. Dey , Endocrinology 2014, 155, 2718. 24823394 10.1210/en.2014-1265 PMC4060188

[34] H. Bayir , T. S. Anthonymuthu , Y. Y. Tyurina , S. J. Patel , A. A. Amoscato , A. M. Lamade , Q. Yang , G. K. Vladimirov , C. C. Philpott , V. E. Kagan , Cell Chem. Biol. 2020, 27, 387. 32275865 10.1016/j.chembiol.2020.03.014 PMC7218794

[35] J. R. Requena , M. X. Fu , M. U. Ahmed , A. J. Jenkins , T. J. Lyons , J. W. Baynes , S. R. Thorpe , Biochem. J. 1997, 322, 317. 9078279 10.1042/bj3220317 PMC1218194

[36] G. Barrera , S. Pizzimenti , M. Daga , C. Dianzani , A. Arcaro , G. P. Cetrangolo , G. Giordano , M. A. Cucci , M. Graf , F. Gentile , Antioxidants 2018, 7, 102. 30061536 10.3390/antiox7080102 PMC6115986

[37] J. Yuan , S. Aikawa , W. Deng , A. Bartos , G. Walz , F. Grahammer , T. B. Huber , X. Sun , S. K. Dey , Nat. Commun. 2019, 10, 5425. 31780662 10.1038/s41467-019-13489-4 PMC6882879

[38] K. S. Fritz , D. R. Petersen , Chem. Res. Toxicol. 2011, 24, 1411. 21812433 10.1021/tx200169n PMC3178011

[39] M. Akagawa , Free Radic. Res. 2021, 55, 307. 33183115 10.1080/10715762.2020.1851027

[40] X. Sun , A. Bartos , J. A. Whitsett , S. K. Dey , Mol. Endocrinol. 2013, 27, 1492. 23885093 10.1210/me.2013-1086 PMC3753427

[41] T. Hiraoka , Y. Hirota , Y. Fukui , M. Gebril , T. Kaku , S. Aikawa , T. Hirata , S. Akaeda , M. Matsuo , H. Haraguchi , M. Saito‐Kanatani , R. Shimizu‐Hirota , N. Takeda , O. Yoshino , T. Fujii , Y. Osuga , Sci. Rep. 2020, 10, 15523. 32968170 10.1038/s41598-020-72640-0 PMC7511330

[42] I. Dalle‐Donne , D. Giustarini , R. Colombo , R. Rossi , A. Milzani , Trends Mol. Med. 2003, 9, 169. 12727143 10.1016/s1471-4914(03)00031-5

[43] T. Nyström , EMBO J. 2005, 24, 1311. 15775985 10.1038/sj.emboj.7600599 PMC1142534

[44] B. Chu , N. Kon , D. Chen , T. Li , T. Liu , L. Jiang , S. Song , O. Tavana , W. Gu , Nat. Cell Biol. 2019, 21, 579. 30962574 10.1038/s41556-019-0305-6 PMC6624840

[45] S. Gupta , A. Agarwal , R. K. Sharma , Obstet. Gynecol. Surv. 2005, 60, 807. 16359563 10.1097/01.ogx.0000193879.79268.59

[46] S. Gupta , N. Aziz , L. Sekhon , R. Agarwal , G. Mansour , J. Li , A. Agarwal , Obstet. Gynecol. Surv. 2009, 64, 750. 19849867 10.1097/OGX.0b013e3181bea0ac

[47] O. Beharier , V. A. Tyurin , J. P. Goff , J. Guerrero‐Santoro , K. Kajiwara , T. Chu , Y. Y. Tyurina , C. M. St Croix , C. T. Wallace , S. Parry , W. T. Parks , V. E. Kagan , Y. Sadovsky , Proc Natl Acad Sci 2020, 117, 27319. 33087576 10.1073/pnas.2009201117 PMC7959495

[48] A. M. Hantak , I. C. Bagchi , M. K. Bagchi , Int. J. Dev. Biol. 2014, 58, 139. 25023679 10.1387/ijdb.130348mb PMC4768910

[49] S. Zhang , S. Kong , B. Wang , X. Cheng , Y. Chen , W. Wu , Q. Wang , J. Shi , Y. Zhang , S. Wang , J. Lu , J. P. Lydon , F. Demayo , W. S. Pear , H. Han , H. Lin , L. Li , H. Wang , Y.‐L. Wang , B. Li , Q. Chen , E. Duan , H. Wang , Cell Res. 2014, 24, 925. 24971735 10.1038/cr.2014.82 PMC4123295

[50] G. Serviddio , G. Loverro , M. Vicino , F. Prigigallo , I. Grattagliano , E. Altomare , G. Vendemiale , J. Clin. Endocrinol. Metab. 2002, 87, 2843. 12050261 10.1210/jcem.87.6.8543

[51] M. Fedorova , R. C. Bollineni , R. Hoffmann , Mass Spectrom. Rev. 2014, 33, 79. 23832618 10.1002/mas.21381

[52] S. Jin , Proc. Natl. Acad. Sci. 2019, 116, 6848. 30872480 10.1073/pnas.1814597116 PMC6452687

[53] L. Magtanong , G. D. Mueller , K. J. Williams , M. Billmann , K. Chan , D. A. Armenta , L. E. Pope , J. Moffat , C. Boone , C. L. Myers , J. A. Olzmann , S. J. Bensinger , S. J. Dixon , Cell Chem. Biol. 2022, 29, 1409. 35809566 10.1016/j.chembiol.2022.06.004 PMC9481678

[54] Q.‐Z. Tuo , Y. Liu , Z. Xiang , H.‐F. Yan , T. Zou , Y. Shu , X.‐L. Ding , J.‐J. Zou , S. Xu , F. Tang , Y.‐Q. Gong , X.‐L. Li , Y.‐J. Guo , Z.‐Y. Zheng , A.‐P. Deng , Z.‐Z. Yang , W.‐J. Li , S.‐T. Zhang , S. Ayton , A. I. Bush , H. Xu , L. Dai , B. Dong , P. Lei , Signal Transduct Target Ther. 2022, 7, 59. 35197442 10.1038/s41392-022-00917-z PMC8866433

[55] Y. Wang , M. Zhang , R. Bi , Y. Su , F. Quan , Y. Lin , C. Yue , X. Cui , Q. Zhao , S. Liu , Y. Yang , D. Zhang , Q. Cao , X. Gao , Redox Biol. 2022, 51, 102262. 35180475 10.1016/j.redox.2022.102262 PMC8857079

[56] Y. Li , D. Feng , Z. Wang , Y. Zhao , R. Sun , D. Tian , D. Liu , F. Zhang , S. Ning , J. Yao , X. Tian , Cell Death Differ. 2019, 26, 2284. 30737476 10.1038/s41418-019-0299-4 PMC6889315

[57] C. A. Hubel , J. M. Roberts , R. N. Taylor , T. J. Musci , G. M. Rogers , M. K. Mclaughlin , Am. J. Obstet. Gynecol. 1989, 161, 1025. 2679100 10.1016/0002-9378(89)90778-3

[58] T. L. Duarte , J. V. Neves , J Vis Exp 2022, 179, e63469. 10.3791/63469 35156663

[59] Y. Yu , L. Jiang , H. Wang , Z. Shen , Q. Cheng , P. Zhang , J. Wang , Q. Wu , X. Fang , L. Duan , S. Wang , K. Wang , P. An , T. Shao , R. T. Chung , S. Zheng , J. Min , F. Wang , Blood 2020, 136, 726. 32374849 10.1182/blood.2019002907 PMC7414596

[60] S. Van Coillie , E. Van San , I. Goetschalckx , B. Wiernicki , B. Mukhopadhyay , W. Tonnus , S. M. Choi , R. Roelandt , C. Dumitrascu , L. Lamberts , G. Dams , W. Weyts , J. Huysentruyt , B. Hassannia , I. Ingold , S. Lele , E. Meyer , M. Berg , R. Seurinck , Y. Saeys , A. Vermeulen , A. L. N. van Nuijs , M. Conrad , A. Linkermann , M. Rajapurkar , P. Vandenabeele , E. Hoste , K. Augustyns , T. Vanden Berghe , Nat. Commun. 2022, 13, 1046. 35210435 10.1038/s41467-022-28718-6 PMC8873468

[61] X. Chen , R. Kang , G. Kroemer , D. Tang , Nat. Rev. Clin. Oncol. 2021, 18, 280. 33514910 10.1038/s41571-020-00462-0

[62] J. Yuan , Y. Zhang , Y. Sheng , X. Fu , H. Cheng , R. Zhou , Autophagy 2015, 11, 1081. 26060891 10.1080/15548627.2015.1040970 PMC4590641

