# Supplementary material for: Excessive Lipid Peroxidation in Uterine Epithelium Causes Implantation Failure and Pregnancy Loss
Source: Adv Sci (Weinh). 2023 Dec 3;11(4):2302887. doi: 10.1002/advs.202302887 (PMC10811501; doi:10.1002/advs.202302887)
Supplement: Supplementary file 1 — Supporting Information [file ADVS-11-2302887-s001.pdf]

ADVANCED SCIENCE
Open Access

Supporting Information

for *Adv. Sci.*, DOI 10.1002/advs.202302887

Excessive Lipid Peroxidation in Uterine Epithelium Causes Implantation Failure and Pregnancy Loss

Yafang Lu, Yuhan Shao, Weiwei Cui, Zhaoyu Jia, Qian Zhang, Qing Zhao, Zi-Jiang Chen, Junhao Yan, Bo Chu* and Jia Yuan**

Supporting Information

Excessive lipid peroxidation in uterine epithelium causes implantation failure and pregnancy loss

Yafang Lu[1,#], Yuhan Shao[2,3,#], Weiwei Cui[4,#], Zhaoyu Jia[1], Qian Zhang[2,3], Qing Zhao[2,3], Zi-Jiang Chen[2,3], Junhao Yan[2,3,*], Bo Chu[4,*], Jia Yuan[1,*]

Email: Jia Yuan (Jia.Yuan@sdu.edu.cn); Bo Chu (chubo123@sdu.edu.cn); Junhao Yan (yanjunhao@sdu.edu.cn)

Supporting Information includes:

Supplementary Figures 1-10

Supplementary Tables 1-4

Supplementary Figures

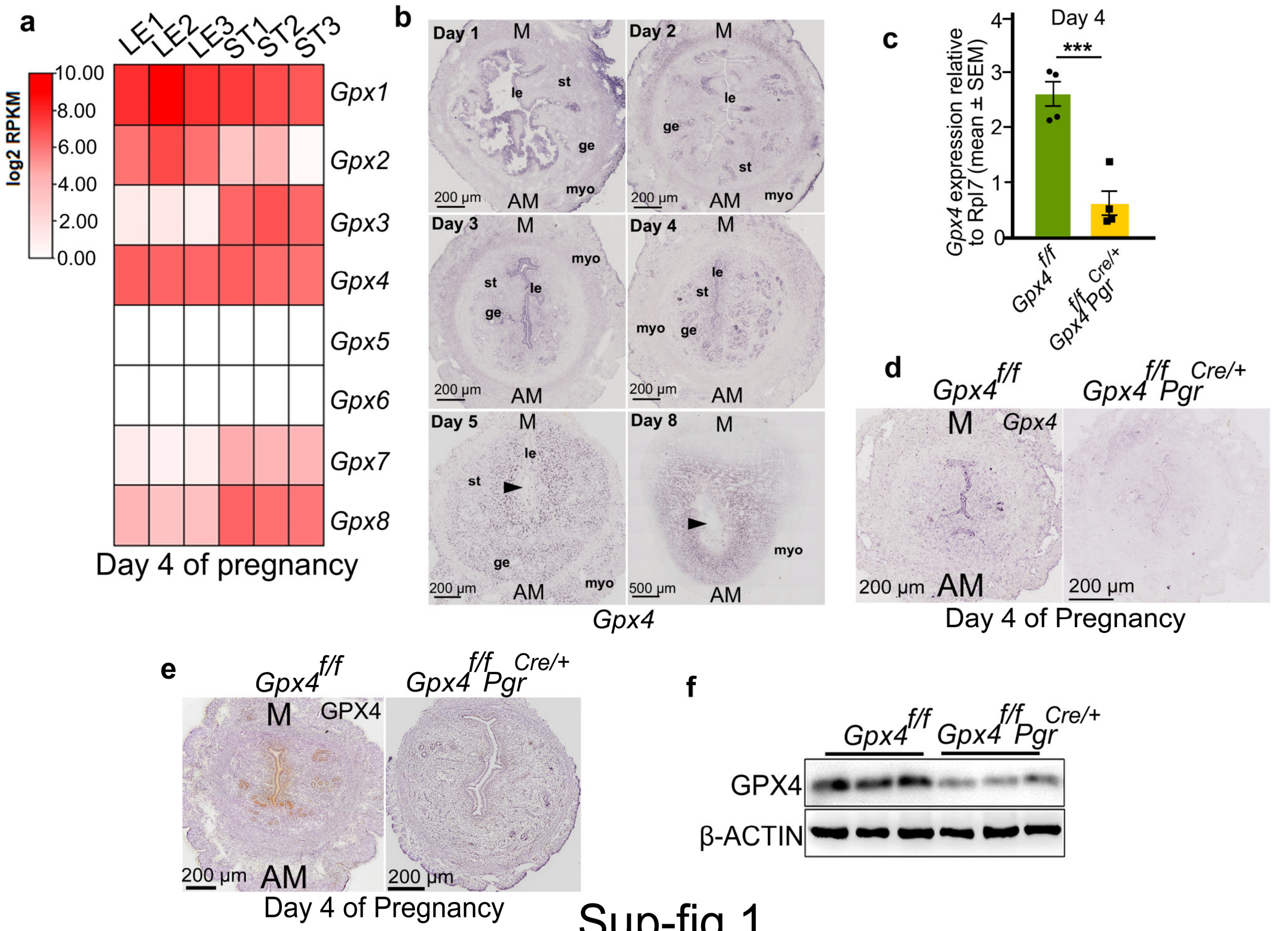

Sup-fig.1

Figure S1. *Gpx4* is expressed in the peri-implantation uterus, and *Gpx4* was efficiently deleted in the uteri of *Gpx4*$^{f/f}$*Pgr*$^{Cre/+}$ mice.

a) Heatmap of RNA-seq analysis of *Gpx* family members' mRNAs (log2 RPKM) in separated epithelial (LE) and stromal cells (ST) from day 4 pregnant uteri (n = 3). RPKM reads per kilobase per million.

b) In situ hybridization (ISH) of *Gpx4* on days 1–5 and 8 of pregnancy. Arrowheads point to the location of embryos. M, mesometrial pole; AM, antimesometrial pole; le, luminal epithelium; ge, glandular epithelium; st, stroma; myo, myometrium. Scale bars, 200 μm (days 1–5) and 500 μm (day 8). Arrowheads indicate the location of the embryos.

c) Quantitative real-time PCR of *Gpx4* in *Gpx4*$^{f/f}$ and *Gpx4*$^{f/f}$*Pgr*$^{Cre/+}$ uteri on day 4 of pregnancy. Data are presented as mean ± SEM (n = 4 mice per group). ***P < 0.001, by two-tailed Student's *t*-test.

d) and **e**) ISH of *Gpx4* and IHC of GPX4 in day 4 uteri of *Gpx4*$^{f/f}$ and *Gpx4*$^{f/f}$*Pgr*$^{Cre/+}$ mice. Scale bars, 200 μm.

f) Western blotting of GPX4 in day 4 uteri of *Gpx4*$^{f/f}$ and *Gpx4*$^{f/f}$*Pgr*$^{Cre/+}$ mice. β-Actin was used as a loading control.

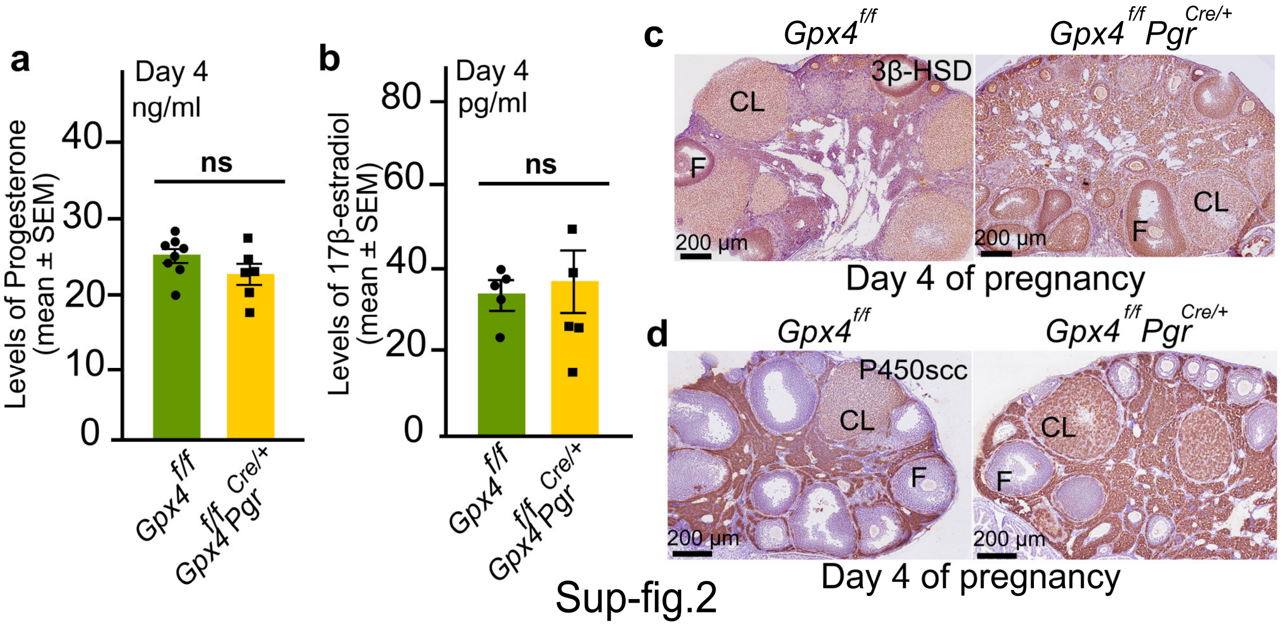

Figure S2. ***Gpx4$^{f/f}$* and *Gpx4$^{f/f}$Pgr$^{Cre/+}$* mice have comparable ovarian steroid hormone levels.**

a) and **b**) Serum progesterone levels and 17β-estradiol levels on day 4 of pregnancy of *Gpx4$^{f/f}$* and *Gpx4$^{f/f}$Pgr$^{Cre/+}$* females. Data are presented as mean ± SEM (n >5 mice per group). ns: not significant, by two-tailed Student's *t*-test.

c) and **d**) IHC of 3β-HSD and P450scc in ovaries from *Gpx4$^{f/f}$* and *Gpx4$^{f/f}$Pgr$^{Cre/+}$* females on day 4 of pregnancy. Scale bars, 200 µm. CL, corpus luteum; F, follicle.

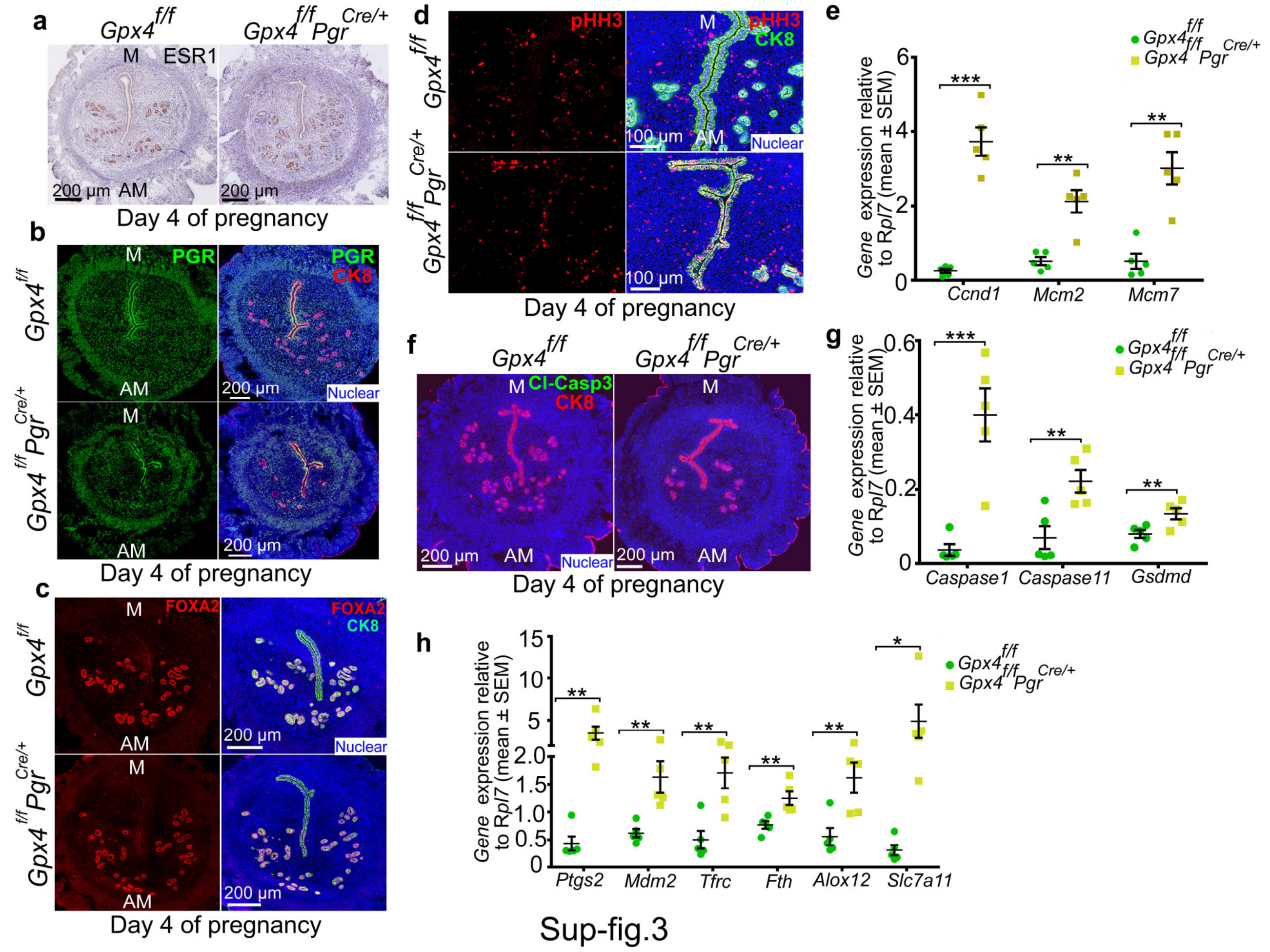

Figure S3. *Gpx4* deficiency impairs uterine receptivity.

a) and **b**) ESR1 and PR location patterns are comparable between *Gpx4*$^{f/f}$ and *Gpx4*$^{f/f}$*Pgr*$^{Cre/+}$ uteri on day 4 of pregnancy. Scale bars, 200 μm.

c) IF of FOXA2 and CK8 in day 4 uteri of *Gpx4*$^{f/f}$ and *Gpx4*$^{f/f}$*Pgr*$^{Cre/+}$ mice. CK8 served as an epithelial marker. Scale bars, 200 μm.

d) IF of PHH3 and CK8 in day 4 uteri of *Gpx4*$^{f/f}$ and *Gpx4*$^{f/f}$*Pgr*$^{Cre/+}$ mice. Scale bars, 100 μm.

e) Quantitative real-time PCR of *Ccnd1, Mcm2, and Mcm7* reveal *Gpx4*$^{f/f}$*Pgr*$^{Cre/+}$ mice have aberrant epithelial cell proliferation on day 4 of pregnancy (n = 5 mice per group).

f) IF of Cl-Casp3 and CK8 indicates no cell apoptosis in day 4 uteri of *Gpx4*$^{f/f}$*Pgr*$^{Cre/+}$ mice. Scale bars, 200 μm.

g) and **h**) Quantitative real-time PCR of relative cell death markers in day 4 uteri of *Gpx4*$^{f/f}$ and *Gpx4*$^{f/f}$*Pgr*$^{Cre/+}$ mice (n = 5 mice per group).

In (e), (g-h), data are presented as mean ± SEM. *P < 0.05, **P < 0.01 and ***P < 0.001, by two-tailed Student's *t*-test.

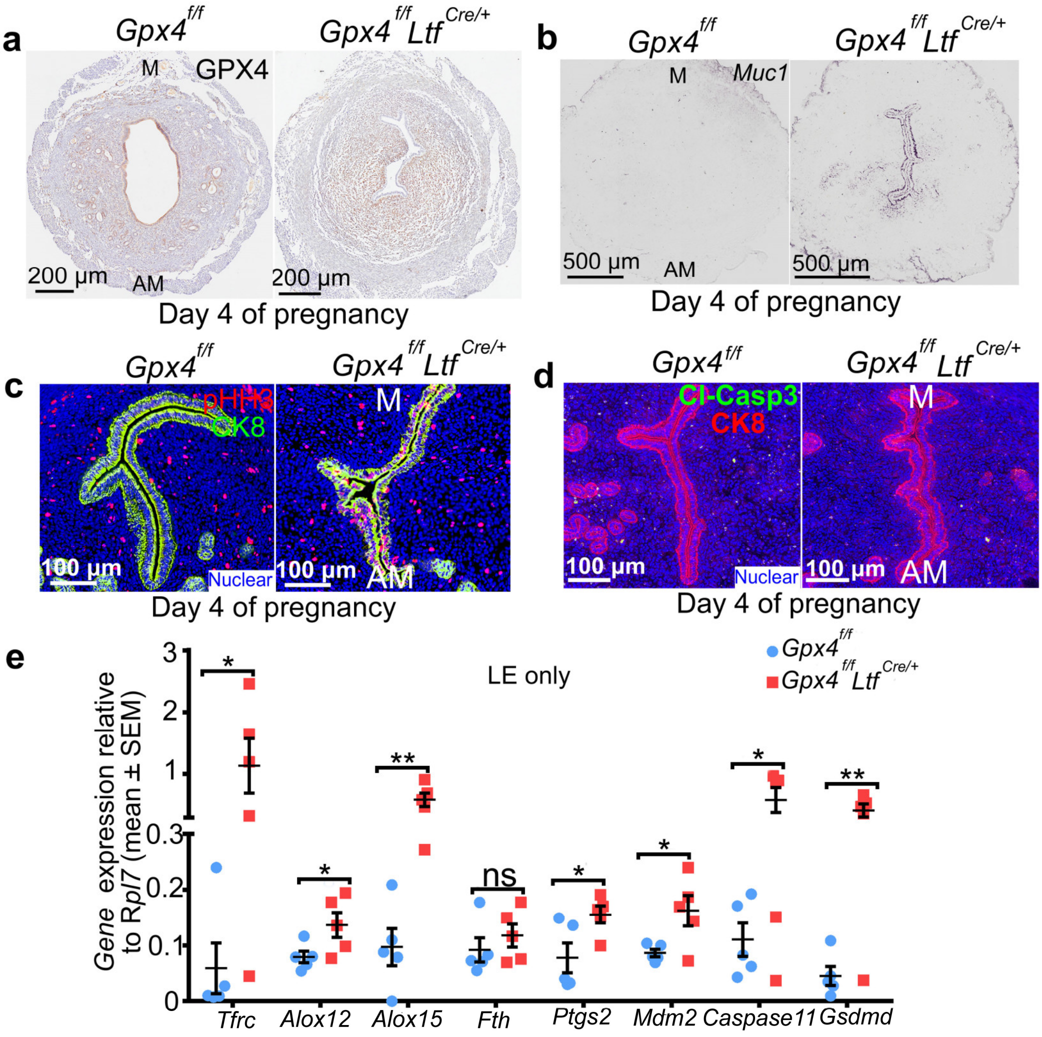

Sup-fig.4

Figure S4. Ablation of *Gpx4* in the uterine epithelium derails uterine receptivity.

a) IHC of GPX4 shows the efficiency of GPX4 deletion in uterine epithelium of *Gpx4*$^{f/f}$*Ltf*$^{Cre/+}$ mice. Scale bars, 200 µm.

b) In situ hybridization of *Muc1* in day 4 pregnant uteri from *Gpx4*$^{f/f}$ and *Gpx4*$^{f/f}$*Ltf*$^{Cre/+}$ mice. Scale bars, 500 µm.

c) IF of PHH3 and CK8 in day 4 uteri of *Gpx4*$^{f/f}$ and *Gpx4*$^{f/f}$*Ltf*$^{Cre/+}$ mice. Scale bars, 100 µm. CK8 served as an epithelial marker.

d) IF of Cl-Casp3 and CK8 in day 4 uteri of *Gpx4*$^{f/f}$ and *Gpx4*$^{f/f}$*Ltf*$^{Cre/+}$ mice. Scale bars, 100 µm.

e) Quantitative real-time PCR of relative cell death markers in separated epithelial cells from day 4 uteri of *Gpx4*$^{f/f}$ and *Gpx4*$^{f/f}$*Ltf*$^{Cre/+}$ mice. Data are presented as mean ± SEM (n = 5 mice per group). $^{*}P < 0.05$ and $^{**}P < 0.01$, ns: not significant, by two-tailed Student's *t*-test.

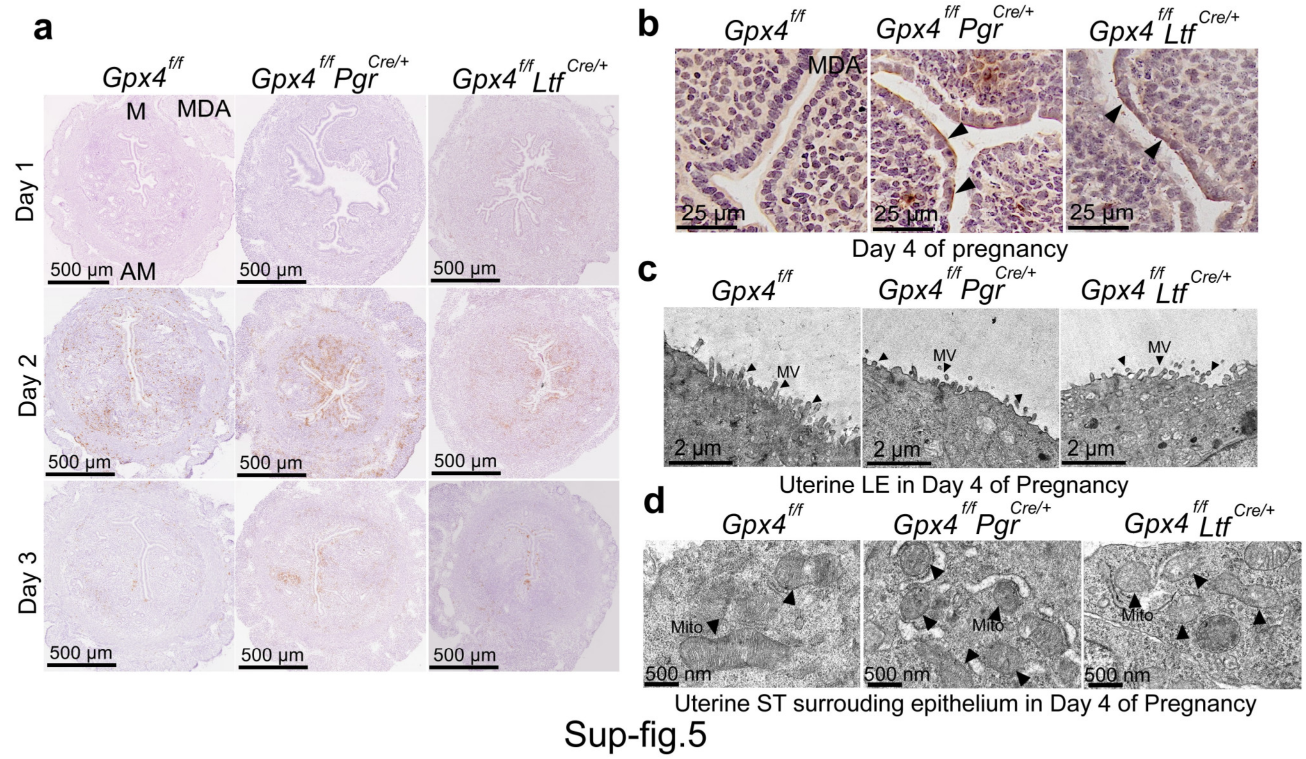

Figure S5. *Gpx4* deletion in uterus causes abnormal redox signaling.

a) IHC of MDA in *Gpx4*$^{f/f}$, *Gpx4*$^{f/f}$*Pgr*$^{Cre/+}$, and *Gpx4*$^{f/f}$*Ltf*$^{Cre/+}$ uteri on days 1-3 of pregnancy. Scale bars, 500 μm.

b) IHC of MDA in *Gpx4*$^{f/f}$, *Gpx4*$^{f/f}$*Pgr*$^{Cre/+}$, and *Gpx4*$^{f/f}$*Ltf*$^{Cre/+}$ uteri on day 4 of pregnancy. Arrowheads point to MDA signaling on the apical surface of uterine epithelium. Scale bars, 25 μm.

c) Transmission EM images of the apical surface of uterine luminal epithelium of *Gpx4*$^{f/f}$, *Gpx4*$^{f/f}$*Pgr*$^{Cre/+}$, and *Gpx4*$^{f/f}$*Ltf*$^{Cre/+}$ mice on day 4 of pregnancy. MV, microvilli. Scale bars, 2 μm.

d) Representative transmission EM images of the stromal cell surrounding luminal epithelium of *Gpx4*$^{f/f}$, *Gpx4*$^{f/f}$*Pgr*$^{Cre/+}$, and *Gpx4*$^{f/f}$*Ltf*$^{Cre/+}$ mice on day 4 of pregnancy. Mito, mitochondrion. Scale bars, 500 nm.

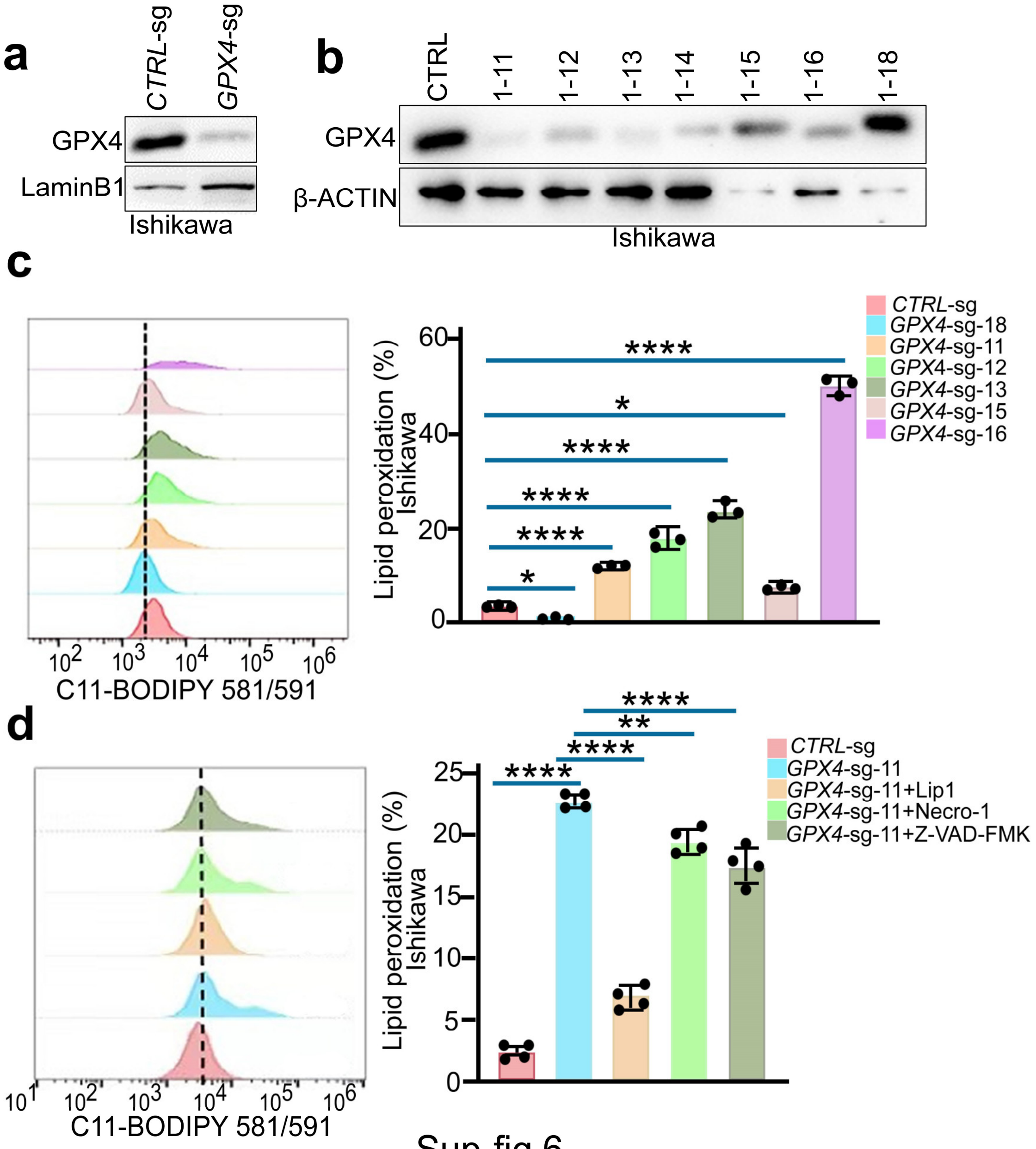

Sup-fig.6

Figure S6. *GPX4* deletion in Ishikawa cells shows increased lipid peroxidation levels.
a) Western blotting of GPX4 in *GPX4* knockout cells. The experiments were repeated twice, independently, with similar results. LaminB1 was used as a loading control.
b) Western blotting of GPX4 in a panel of *GPX4* single Ishikawa crispr colon. Three independent experiments were performed. β-Actin was used as a loading control.
c) Lipid peroxidation levels in *GPX4* single Ishikawa crispr colons were assessed with C11-BODIPY by flow cytometry (n = 3 independent biologically cell cultures).
d) Lipid peroxidation levels in *GPX4* single Ishikawa crispr colon (*GPX4*-sg-11) treated with Lip1, Necro-1, and Z-VAD-FMK (n = 4 independent biologically cell cultures).
In (c-d), data are presented as mean ± SEM. *P < 0.05, **P < 0.01, and ****P < 0.0001, by one-way ANOVA followed by Dunnett's post hoc test.

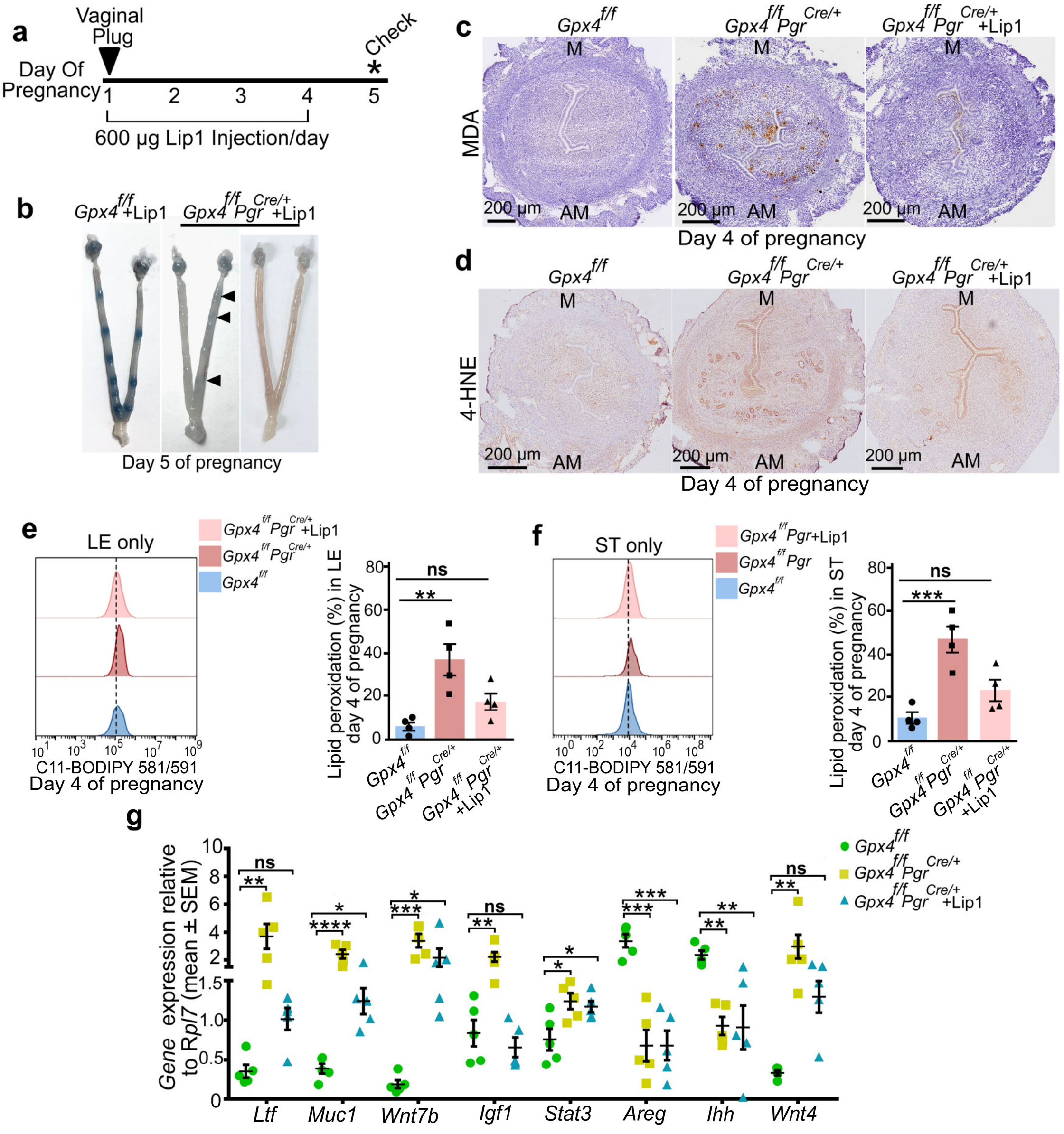

Sup-fig.7

Figure S7. Lipid peroxidation inhibitor Liproxstatin-1 (Lip1) administration effectively decreases the cellular level of lipid peroxide in the uterus on day 4 of pregnancy.

a) Treatment schedule for Lip1 treatment in *Gpx4*$^{f/f}$*Pgr*$^{Cre/+}$ mice.

b) Representative uteri from *Gpx4*$^{f/f}$ and *Gpx4*$^{f/f}$*Pgr*$^{Cre/+}$ mice on day 5 shows defective or failed implantation after Lip1 treatment.

c) and **d**) IHC of MDA and 4-HNE in *Gpx4*$^{f/f}$, *Gpx4*$^{f/f}$*Pgr*$^{Cre/+}$, and *Gpx4*$^{f/f}$*Pgr*$^{Cre/+}$ uteri after Lip1 treatment on day 4 of pregnancy. Scale bars, 200 μm.

e) and **f**) Lipid peroxidation levels in separated uterine LE and stromal cells on day 4 after Lip1 treatment (n = 4 mice per group).

g) Quantitative real-time PCR analysis of receptivity marker genes in *Gpx4*$^{f/f}$*Pgr*$^{Cre/+}$ mice after Lip1 treatment (n = 5 mice per group).

In (e-g), data are presented as mean ± SEM. *P < 0.05, **P < 0.01, ***P < 0.001, and ****P < 0.0001, ns: not significant, by one-way ANOVA followed by Dunnett's post hoc test.

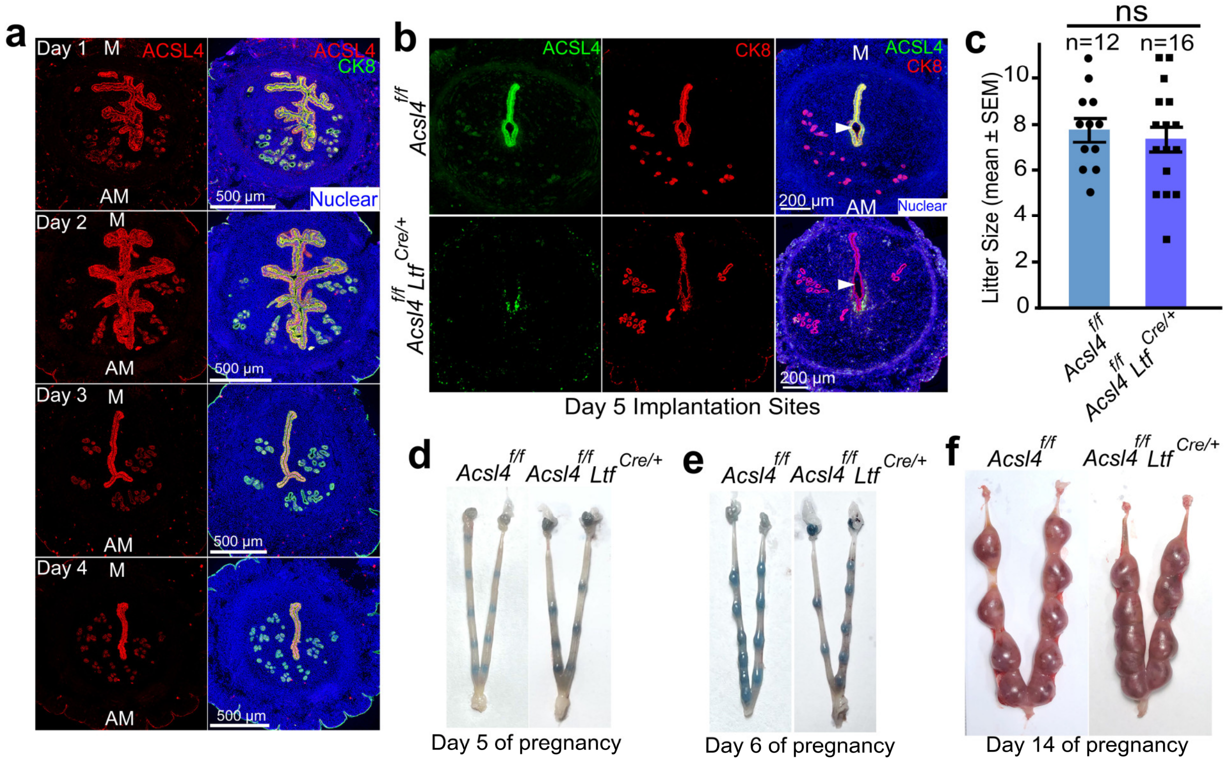

Sup-fig. 8

Figure S8. Inactivation of ACSL4 in uterine epithelium shows normal pregnancy outcomes.

a) IF localization of ACSL4 expression patterns on days 1–4 of pregnancy. Scale bars, 500 μm.

b) IF of ACSL4 and CK8 in day 5 implantation sites of *Acsl4*$^{f/f}$ and *Acsl4*$^{f/f}$*Ltf*$^{Cre/+}$ mice. Scale bars, 200 μm. Arrowheads point to the location of embryos.

c) Pregnancy outcomes in *Acsl4*$^{f/f}$ and *Acsl4*$^{f/f}$*Ltf*$^{Cre/+}$ mice, the litter sizes are comparable between the floxed and *Acsl4*$^{f/f}$*Ltf*$^{Cre/+}$ mice. The number above the bar indicates the number of mice tested. Data are presented as mean ± SEM. ns: not significant, by two-tailed Student's *t*-test.

d-f) Representative uteri from *Acsl4*$^{f/f}$ and *Acsl4*$^{f/f}$*Ltf*$^{Cre/+}$ mice on day 5, day 6, and day 14 of pregnancy.

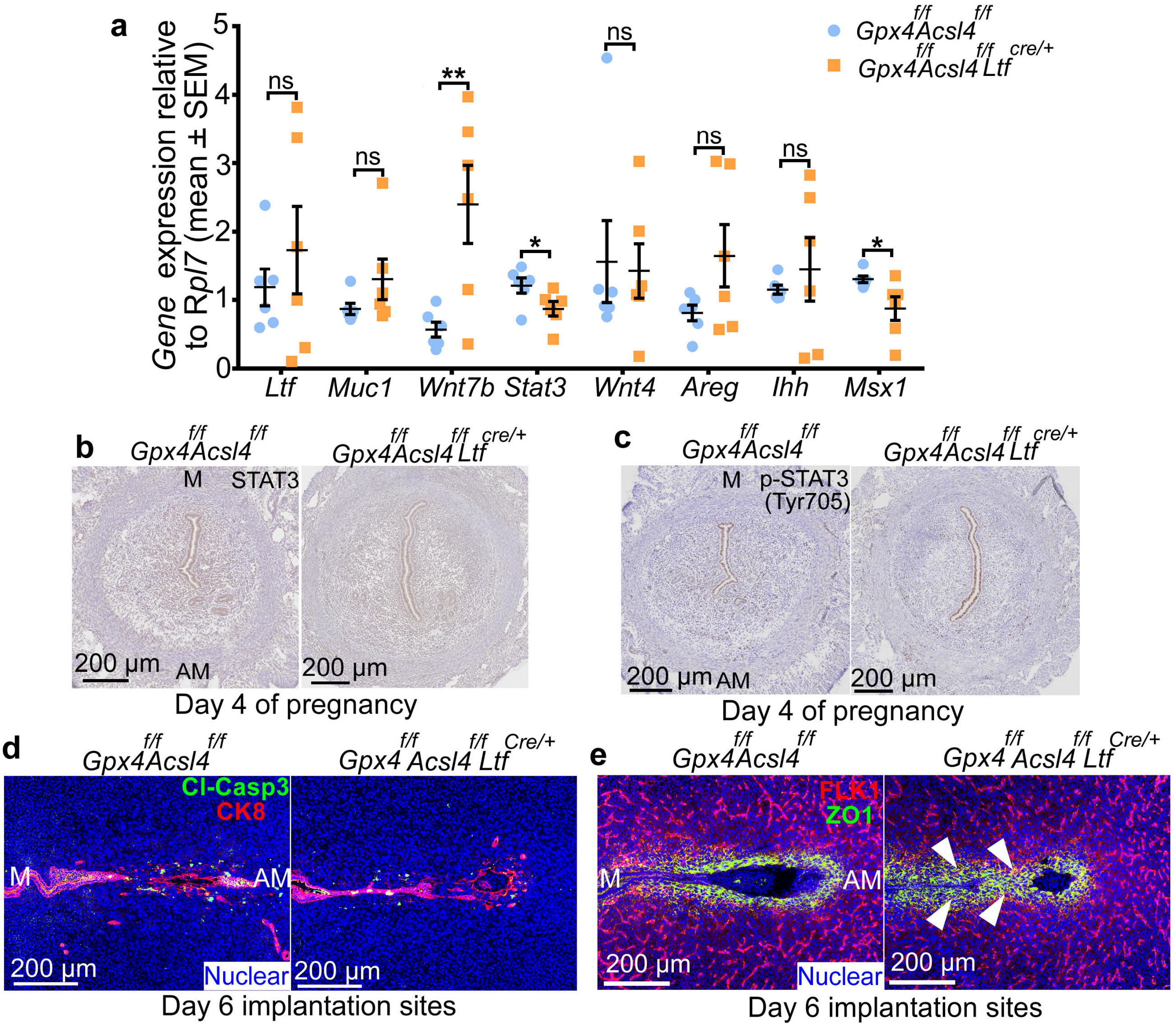

Sup-Fig. 9

Figure S9. Compromised receptivity and aberrant implantation sites in *Gpx4$^{f/f}$Acsl4$^{f/f}$Ltf$^{Cre/+}$* mice.

a) Quantitative real-time PCR analysis of receptivity marker genes in *Gpx4$^{f/f}$Acsl4$^{f/f}$* and *Gpx4$^{f/f}$Acsl4$^{f/f}$Ltf$^{Cre/+}$* uteri on day 4 of pregnancy. Data are reported as means ± SEM (n = 6 mice per group). ns, not significant, *P < 0.05 and **P < 0.01, by two-tailed Student's *t*-test.

b) IHC of STAT3 in *Gpx4$^{f/f}$Acsl4$^{f/f}$* and *Gpx4$^{f/f}$Acsl4$^{f/f}$Ltf$^{Cre/+}$* uteri on day 4 of pregnancy. Scale bars, 200 μm.

c) IHC of p-STAT3 (Tyr705) in *Gpx4$^{f/f}$Acsl4$^{f/f}$* and *Gpx4$^{f/f}$Acsl4$^{f/f}$Ltf$^{Cre/+}$* uteri on day 4 of pregnancy. Scale bars, 200 μm.

d) IF of Cl-Casp3 and CK8 in *Gpx4$^{f/f}$Acsl4$^{f/f}$* and *Gpx4$^{f/f}$Acsl4$^{f/f}$Ltf$^{Cre/+}$* mice on day 6 implantation sites. Scale bars, 200 μm.

e) IF of FLK1 and ZO1 in *Gpx4$^{f/f}$Acsl4$^{f/f}$* and *Gpx4$^{f/f}$Acsl4$^{f/f}$Ltf$^{Cre/+}$* mice on day 6 implantation sites. Arrowheads indicate the invasion of blood vessels into the primary decidual zone in *Gpx4$^{f/f}$Acsl4$^{f/f}$Ltf$^{Cre/+}$* mice. Scale bars, 200 μm.

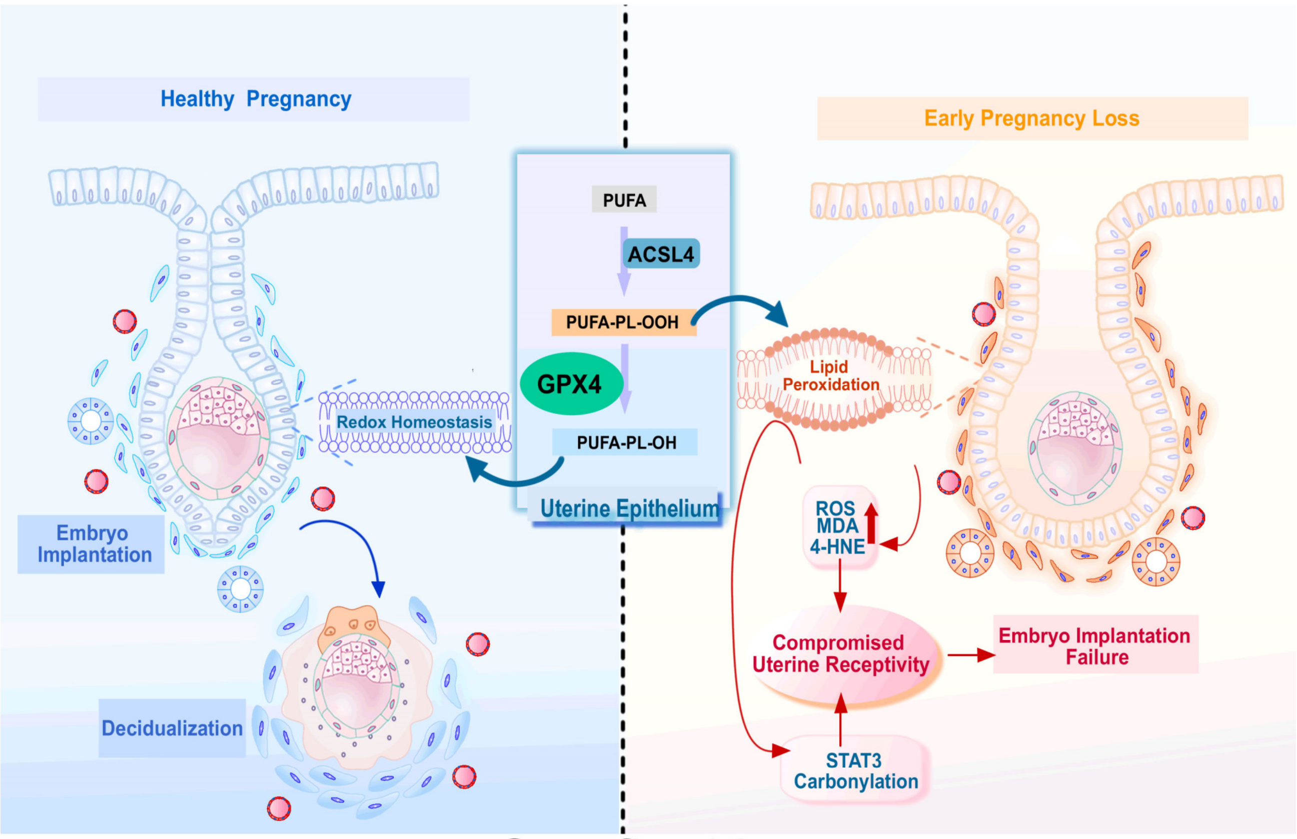

Sup fig-10

Figure S10. A proposed scheme of redox homeostasis's role in the uterine epithelium during pre-implantation.

ACSL4 promotes biosynthesis of polyunsaturated phospholipids in the uterine epithelium, GPX4 acts as a phospholipid hydroperoxidase to convert PL-OOH to PL-OH, which serves to regulate lipid peroxides levels. Excessive lipid peroxides cause STAT3 carbonylation and result in aberrant uterine receptivity, leading to implantation failure.

Supplementary Tables

Table S1. Clinical parameters of serum donors of successful pregnancy and recurrent implantation failure patients undertaken IVF treatment

	Controls (n=44)	RIFs (n=33)	P
Age (years)	32.07±0.77	33.79±0.66	0.1091
Infertility duration (years)	3.37±0.35	5.30±0.54	0.0026
BMI (kg/m^2)	24.65±0.61	24.00±0.57	0.4529
Gravida, median (25th, 75th)	1 (0, 2)	0 (0, 2)	0.8811
Parity, median (25th, 75th)	0 (0, 1)	0 (0, 0)	0.9838
Basal FSH (IU/L)	6.62±0.41	6.31±0.30	0.5776
Basal LH (IU/L)	4.67±0.34	5.35±0.53	0.2612
Right AFC	6.54±0.46	6.52±0.51	0.7439
Left AFC	6.07±0.49	5.46±0.52	0.3986
Embryo transfer No.	2 (1, 2)	2 (1, 2)	> 0.9999
Implantation failure times, median (25th, 75th)	0 (0, 0)	4 (3, 4)	< 0.0001

FSH, follicle-stimulating hormone; LH, luteinizing hormone; AFC, antral follicle count

These non-normally distributed clinical parameters data are presented as medians and quartiles, and statistically analyzed using a non-parametric Kolmogorov-Smirnov test.

Table S2. Clinical parameters of endometrium donors of successful pregnancy and recurrent implantation failure patients undertaken IVF treatment

Patients No.	Age (years)	Infertility duration (years)	BMI (kg/m^2)	Menstrual cycle (days)	Right AFC	Left AFC	Basal FSH (IU/L)	Basal LH (IU/L)	Basal E2 (pg/ml)	Basal P4 (ng/ml)	Basal PRL (ng/ml)	Basal T (ng/dl)	TSH (uIU/mL)	Hysteroscopy
N1	37	14	23.05	30	8	7	5.98	3.11	36.4	0.75	20.1	20.41	1.84	E
N2	37	6	23.81	28	6	7	8.57	3.42	17.6	0.19	12.23	7.08	1.35	N
N3	28	5	23.6	31	10	4	5.65	4.57	22.6	0.18	21.12	32.01	1.28	N
N4	36	9	19.16	26	4	4	6.76	4.51	32	0.22	21.38	26.73	1.563	N
N5	32	6	26.06	40	15	14	5.28	10.09	32.51	0.26	14	27.67	1.24	N
N6	34	1	18.17	32	7	5	6.76	4.69	31.8	0.14	9.99	23.5	1.53	N
N7	37	7	19.53	30	2	5	7.55	4.31	20.7	0.23	16.9	9.43	2.57	N
N8	32	4	21.88	30	5	8	7.969	4.549	37	0.5	17.51	27	1.408	N
F1	24	1	22.15	30	8	6	2.64	4.48	30.8	0.21	23.68	22.83	1.63	N
F2	35	9	22.05	40	14	9	6.32	5.63	29.6	0.27	11.7	59.72	2.17	N
F3	36	5	27.33	30	9	7	9.33	4.56	23.6	0.12	9.49	16.4	2.83	N
F4	33	12	21.97	32	7	8	7.04	7.03	26.4	0.36	19	19.4	0.701	N
F5	30	7	21.26	40	14	14	4.49	6.39	44.2	0.21	26.64	10.4	3.39	N
F6	40	9	20.63	28	4	6	8.34	8.67	32.1	0.58	4.64	21.29	2.02	N
F7	32	2	20.57	27	4	4	8.85	5.03	43.5	0.17	17	20.9	1.38	N
F8	31	3	21.91	40	13	11	5.52	7.04	33.7	0.2	17.4	27.5	1.46	N
F9	39	3	18.18	31	9	7	6.96	8.05	32.3	0.29	14.76	7.5	1.7	N
F10	36	1	23.32	30	4	6	5.32	10.77	86.52	0.136	24.67	24.5	2.99	N
F11	31	2	22.23	30	7	4	4.86	1.04	28.7	0.09	17.19	2.5	1.48	N
F12	27	3	19.36	29	11	9	6.77	4.09	56	0.54	46.4	37.41	1.66	N
F13	31	5	25.22	28	7	6	5.51	3.02	20	0.21	19.67	26.39	2.45	E
F14	33	3	20.48	30	8	5	6.78	7.46	31.3	0.23	17.49	34.27	3.09	N
F15	32	2	18.92	28	4	7	8.7	5.42	62	0.92	27.31	43	2.05	N
F16	37	1	23.29	31	3	6	5.05	4.49	32.9	0.386	16.76	18.6	1.16	N

E, endometritis; N, normal

Table S3. Antibodies used in this study.

Antibodies	Company	Catalog/clone Number	Dilution	Application
GPX4	Abcam	ab125066	1:200	Immunohistochemistry/ Immunoblot
COX2	Cayman	160106	1:300	Immunofluorescence
ACSL4	Abcam	ab155282	1:200	Immunofluorescence/ Immunoblot
KI67	Invitrogen	14-5698-82	1:500	Immunofluorescence
CTNNB1	CST	8480	1:300	Immunofluorescence
PHH3	CST	9701	1:300	Immunofluorescence
CK8	Emd Millipore	NP-112447	1:1000	Immunofluorescence
FOXA2	Seven Hills Bioreagents	WRAB-1200	1:300	Immunofluorescence
Cl-Caspase3	CST	9661	1:300	Immunofluorescence
ESR1	ZENBIO	R24251	1:300	Immunohistochemistry
PR	CST	8757	1:300	Immunofluorescence
P450-Scc	Abcam	ab272494	1:300	Immunohistochemistry
3β-HSD	Abcam	ab65156	1:300	Immunohistochemistry
MDA	Adipogen	JAI-MMD-030N	1:200	Immunohistochemistry
4HNE	Abcam	ab48506	1:200	Immunohistochemistry/ Immunoblot
STAT3	ZENBIO	251611	1:300	Immunohistochemistry/ Immunoblot
p-STAT3(Tyr705)	ZENBIO	310019	1:200	Immunohistochemistry
FLK1	CST	2479	1:200	Immunofluorescence
ZO-1	Invitrogen	2251979	1:200	Immunofluorescence
p-STAT3 (Tyr705)	ZENBIO	340799	1:1000	Immunoblot
p-STAT3 (Ser727)	ZENBIO	R25804	1:1000	Immunoblot
β-ACTIN	Proteintech	20536-1-AP	1:3000	Immunoblot
Flag	Sigma	F1804	1:1000	Immunoblot/IP
Lamin B1	Santa Cruz	sc-374015	1:1000	Immunoblot
594-conjugated Donkey Anti-Rabbit IgG	Proteintech	SA00013-8	1:500	Immunofluorescence
Alexa fluor 594 Donkey Anti-rat IgG	Invitrogen	A21209	1:500	Immunofluorescence
488-conjugated Donkey Anti-Rabbit IgG	Proteintech	SA00013-6	1:500	Immunofluorescence
Alexa fluor 488 Donkey Anti-Rat IgG	Invitrogen	A-21208	1:500	Immunofluorescence

Goat Anti-Rabbit IgG H&L(HRP)	abcam	ab205718	1:5000	Immunoblot
Goat Anti-Mouse IgG H&L(HRP)	abcam	ab6789	1:5000	Immunoblot

Table S4. Primers used in this study.

Genes	Primers
Gpx4-Mus-F	5'-TTACGAATCCTGGCCTTCC-3'
Gpx4-Mus-R	5'-TGGGCTGGACTTTCATCCAT-3'
Ccnd1-Mus-F	5'-GCCCTCCGTATCTTACT-3'
Ccnd1-Mus-R	5'-GCACTTCTGCTCCTCAC-3'
Mcm2-Mus-F	5'-TGCTGACGCCCTGACCT-3'
Mcm2-Mus-R	5'-CGGCCTCGTAGACATCG-3'
Mcm7-Mus-F	5'-CTGTATGTGGACCTGGAT-3'
Mcm7-Mus-R	5'-GCTTGCTGCTACTTGG-3'
Gsdmd-Mus-F	5'-CTCGCTTCGCTTGGTG-3'
Gsdmd -Mus-R	5'-CCTCTGCTGCCGCTTA-3'
Caspase11-Mus-F	5'-TGCCTTCTACTCTACAACC-3'
Caspase11-Mus-R	5'-CTGGGAATGAATACTTGC-3'
Caspase1-Mus-F	5'-AGACAAGCCCAAGGTG-3'
Caspase1-Mus-R	5'-CATCCGTTAAGAAATCC-3'
Muc1-Mus-F	5'-AGCCCCTATGAGGAGGTTTCG-3'
Muc1-Mus-R	5'-AAGTGGTCACCACAGCTGGG-3'
Ltf-Mus-F	5'-GGTGGCAGAACGAGATG-3'
Ltf-Mus-R	5'-GGTCGCAGTTTGTAGGG-3'
Wnt7b-Mus-F	5'-TGAGGCGGGCAGAAAGG-3'
Wnt7b-Mus-R	5'-CCTGACACACCGTGACACTTA-3'
Msx1-Mus-F	5'-CTTCCTCCTGGTTGTCGCT-3'
Msx1-Mus-R	5'-CTCTTGGCCTCTGCACCCTTAGTTT-3'
Lif-Mus-F	5'-GATGGTCGCATACCTGAG-3'
Lif-Mus-R	5'-GCTGGCAACCCAACTTTT-3'
Igf1-Mus-F	5'-GGTGGATGCTCTTCAGTTC-3'
Igf1-Mus-R	5'-TTTGTAGGCTTCAGTGGG-3'
Hoxa10-Mus-F	5'-CTACGATGCTGCGGACAAA-3'
Hoxa10-Mus-R	5'-CTGCGACAGGCGGAAGT-3'
Areg-Mus-F	5'-GACAAGAAAATGGGACTGTGC-3'
Areg-Mus-R	5'-GGCTTGGCAATGATTCAACT-3'
Ihh-Mus-F	5'-CATCTTCAAGGACGAGGAGAACA-3'
Ihh-Mus-R	5'-CATGACAGAGATGGCCAGTGA-3'
Wnt4-Mus-F	5'-CGAGCAATTGGCTGTACCTG-3'
Wnt4-Mus-R	5'-TCCGGAACTGGTATTGGCAC-3'
Hand2-Mus-F	5'-GCCTCCTTACCCAACTCTT-3'
Hand2-Mus-R	5'-GATAACCGACCCGACAGA-3'
Rpl7-Mus-F	5'-AGCTGGCAACTTCTATGTGC-3'
Rpl7-Mus-R	5'-CGCAGCATGTTAATTGAAGCC-3'
Ptgs2-Mus-F	5'-ATGAGCACAGGATTTGACCA-3'
Ptgs2-Mus-R	5'-TGGGCTTCAGCAGTAATTTG-3'

Mdm2-Mus-F	5'-TGTGAAGGAGCACAGGAAAA-3'
Mdm2-Mus-R	5'-TCCTTCAGATCACTCCCACC -3'
Tfrc-Mus-F	5'-GGATGTTCCTACATCATCTCG-3'
Tfrc-Mus-R	5'-CATCTCGCCAGACTTTGCTGA-3'
Fth-Mus-F	5'-ATCTCATGAGGAGAGGGAGCA-3'
Fth-Mus-R	5'-GCACACTCCATTGCATTCAG-3'
Alox12-Mus-F	5'-GATCACTGAAGTGGGGCTGT-3'
Alox12-Mus-R	5'-CACACATGGTGAGGAAATGG-3'
Slc7a11-Mus-F	5'-AGAGACACAAGTCTAATGGG-3'
Slc7a11-Mus-R	5'-AGGCCACGTTTGTCAGTACGT-3'
Alox15-Mus-F	5'-CGGTCTACTTGTCTCCCTGC-3'
Alox15-Mus-R	5'-ATCCGCTTCAAACAGAGTGC-3'
Stat3-Mus-F	5'-AACAGCCGCCGTAGTGAC-3'
Stat3-Mus-R	5'-GAAATCAAAGTCGTCCTG-3'
GPX4-Homo-F	5'-AAGGACATCGACGGGCACAT-3'
GPX4-Homo-R	5'-ATCTCTTCGTTACTCCCTGG-3'
ACSL4-Homo-F	5'-GATGCACCTCTTTGCAATCTG-3'
ACSL4-Homo-R	5'-GCTCCAACTCTGCCAGTAGTA-3'
ACTIN-Homo-F	5'-GTGCGACGAAGACGAGAC-3'
ACTIN-Homo-R	5'-AATCTTTCTGACCCATACCG-3'
STAT3 (P689A)-F	5'-TGTCGGGCAGAGAGCCAGGAGCATCCTGAAGCTGACCC-3'
STAT3 (P689A)-R	5'-GCTCCTGGCTCTCTGCCCGACAATACTTTCCGAATGCC-3'
